# Gibberellin metabolism in *Vitis vinifera* L. during bloom and fruit-set: functional characterization and evolution of grapevine gibberellin oxidases

**DOI:** 10.1093/jxb/ert251

**Published:** 2013-09-04

**Authors:** Lisa Giacomelli, Omar Rota-Stabelli, Domenico Masuero, Atiako Kwame Acheampong, Marco Moretto, Lorenzo Caputi, Urska Vrhovsek, Claudio Moser

**Affiliations:** ^1^Research and Innovation Centre-Fondazione Edmund Mach, Via E. Mach 1, 38010 S. Michele all’Adige (TN), Italy; ^2^Department of Fruit Tree Sciences, Volcani Center, ARO, Israel

**Keywords:** Anthesis, fruit set, gibberellin 2β-hydroxylase, gibberellin 3β-hydroxylase, gibberellin metabolism, gibberellin oxidase, grapevine, inflorescence, *Vitis vinifera*.

## Abstract

Gibberellins (GAs) are involved in the regulation of flowering and fruit-set in grapes (*Vitis vinifera* L.), but the molecular mechanisms behind this process are mostly unknown. In this work, the family of grapevine GA oxidases involved in the biosynthesis and deactivation of GAs was characterized. Six putative GA 20-oxidase (GA20ox), three GA 3-oxidase (GA3ox), and eight GA 2-oxidase (GA2ox) proteins, the latter further divided into five C_19_-GA 2ox and three C_20_-GA2ox proteins, were identified. Phylogenetic analyses suggest a common origin of the GA3ox and C_19_-GA2ox groups and challenge previous evolutionary models. *In vitro* analysis revealed that all GA3ox and GA20ox enzymes prefer substrates of the non-13-hydroxylation pathway. In addition, ectopic expression of GA2ox genes in *Arabidopsis thaliana* confirmed the activity of their encoded proteins *in vivo*. The results show that bioactive GA_1_ accumulates in opening grapevine flowers, whereas at later developmental stages only GA_4_ is detected in the setting fruit. By studying the expression pattern of the grapevine GA oxidase genes in different organs, and at different stages of flowering and fruit-set, it is proposed that the pool of bioactive GAs is controlled by a fine regulation of the abundance and localization of GA oxidase transcripts.

## Introduction

**Fig. 1.**
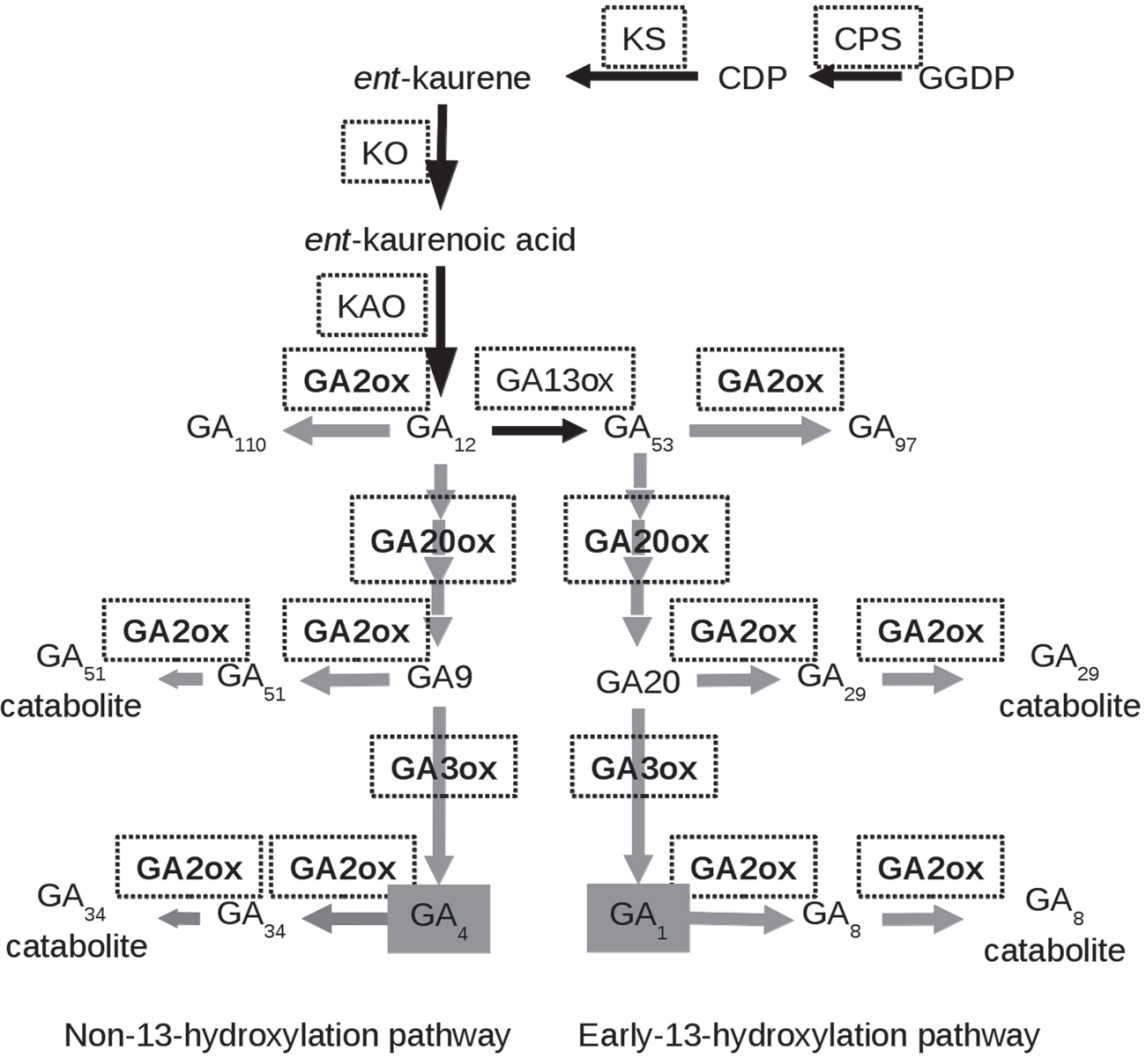
Metabolism of GA_1_ and GA_4_ in plants. Schematic representation of the early-13-hydroxylation and non-13-hydroxylation pathways in plants. Enzymes are indicated in white boxes. Abbreviations: geranylgeranyl diphospate (GGDP), *ent*-copalyl diphosphate (CDP), *ent-*copalyl diphosphate synthase (CPS), *ent*-kaurene synthase (KS), *ent*-kaurene oxidase (KO), *ent-*kaurenoic acid synthase (KAO), GA 13-oxidase (GA13ox), GA 20-oxidase (GA20ox), GA 3-oxidase (GA3ox), and GA 2-oxidase (GA2ox).

Gibberellins (GAs) are a family of plant hormones that promote cell division and elongation in several tissues and are involved in numerous developmental processes, such as flowering and fruit-set. Despite the identification of 136 GA structures in nature (www.plant-hormones.info, last accessed on 23 July 2013) only a handful are biologically active. GA_1_ and GA_4_ are the most common active forms in higher plants, although some species also accumulate GA_3_, GA_5_, and GA_7_ (e.g. spinach and maize). GAs are cyclic diterpenoids derived from *ent*-kaurene, which is first oxidized by membrane-associated mono- oxygenases (*ent*-kaurene oxidase and *ent*-kaurenoic acid oxidase), and subsequently oxidized by soluble 2-oxoglutarate-dependent dioxygenases (2-ODDs). GA_1_ and GA_4_ are formed in plants by two parallel pathways named the early-13-hydroxylation and non-13-hydroxylation pathways, respectively, and illustrated in Fig. 1. The mono-oxygenases convert *ent*-kaurene into GAs with a C_20_ carbon skeleton, GA_12_ and GA_53_, which are converted to the C_19_ products GA_9_ and GA_20_, respectively, through the sequential oxidation of C_20_ by GA 20-oxidase (GA20ox) proteins. These intermediates are oxidized to the biologically active GA_4_ and GA_1_ by GA 3-oxidase (GA3ox). The pool of active GAs is maintained by controlling their biosynthesis as well as their deactivation, mainly through 2β-hydroxylation, but also by conjugation with sugars (Schneider and Schliemann, 1994), by methylation (Varbanova *et al.*, 2007), and by epoxidation of the 16,17-double bond (Zhu *et al.*, 2006).

GA 2β-hydroxylation is carried out by an additional class of dioxygenases, GA 2-oxidase (GA2ox) proteins, which form two divergent groups in relation to their substrate specificity for C_19_- or C_20_-GAs (C_19_-GA2ox and C_20_-GA2ox, respectively). C_20_-GA2ox proteins have so far been characterized in *Arabidopsis*, spinach, and cucumber (Schomburg *et al.*, 2003; Lee and Zeevaart, 2005; Pimenta Lange *et al.*, 2013). According to Lee and Zeevaart (2005), the *Arabidopsis* C_20_-GA2ox proteins possess a characteristic domain absent in C_19_- GA2ox. However, Pimenta Lange *et al.* (2013) showed that cucumber CsGA2ox4 is phylogenetically close to the C_19_-GA2ox, but possesses both C_19_- and C_20_-GA2ox activity, making the functional distinction less clear.

In many plant species, the GA20ox, GA3ox, and GA2ox functions are carried out by enzymes encoded by small gene families (Phillips *et al.*, 1995; Thomas *et al.*, 1999; Sakamoto *et al.*, 2004; Han and Zhu, 2011), which account for some functional redundancy but also for tissue specificity (Mitchum *et al.*, 2006).

The control of the GA biosynthetic pathway is mainly exerted on GA oxidases through feedback loop mechanisms (Middleton *et al.*, 2012) and by localization of their expression to limited tissues. Active GA synthesis restricted to the zone of expansion of the xylem ensures proper wood formation in aspen (Israelsson *et al.*, 2005), whereas distinct localization of different classes of GA2ox ensures distinct function in shoot growth and root development in poplar (Gou *et al.*, 2011).

Alteration of the expression levels of GA oxidases has been proven successful in controlling plant stature of both model (Koornneef and Veen, 1980; Huang *et al.*, 1998) and crop species, and often affects several traits. Rice overexpressing *OsGA20ox1* showed an elongated phenotype (Oikawa *et al.*, 2004), whereas plants overexpressing *OsGA2ox1* were dwarf and displayed inhibited cell elongation, delayed flowering, and impairment in the development of reproductive organs (Sakamoto *et al.*, 2001). Tobacco plants engineered to express *AtGA20ox* or *AtGA2ox* ectopically were, respectively, elongated or stunted, and the alteration of the active GA pool affected lignin deposition in these plants (Biemelt *et al.*, 2004). Overexpression of *PtaGA2ox1* in poplar produced a short and stout phenotype (Busov *et al.*, 2003), whereas a dwarf plum hybrid showed enhanced expression of *PslGA2ox* (El-Sharkawy *et al.*, 2012).

Bunch morphology and berry size are economically important traits for several grape varieties and are often controlled with the aid of GA applications (Weaver, 1961; Khurshid *et al.*, 1992; Dokoozlian and Peacock, 2001). In wine varieties, GA treatment is performed at bloom (anthesis) to achieve loosening and better aeration of the bunches, rendering them less susceptible to fungal rot, whereas in table varieties treatment is often performed after fruit-set mainly to increase berry size.

In grapevine, anthesis coincides with the falling of calyptras, cap structures formed by four petals, which detach at the base of the flower to release carpel and stamens. Following pollination, fruit growth is driven by an initial rapid cell division and then cell expansion together with embryo development (Ojeda *et al.*, 1999). In many plant species, key regulators of these first developmental stages are auxins and GAs produced by the embryo and the surrounding tissues (Ozga and Reinecke, 2003; Hu *et al.*, 2008, Dorcey *et al.*, 2009).

Despite their importance in viticulture, little is known about GA biosynthesis, signal transduction, and regulation of development in vine, and reports on GA measurements are scarce. The content of endogenous bioactive GAs in setting fruits is minimal right past anthesis and increases after 10–20 d (Pérez *et al.*, 2000). A more recent study determined GA concentrations in leaves and internodes (Boss *et al.*, 2003), but did not include information regarding flowers and anthesis. A grapevine GA-insensitive mutant is dwarf and develops inflorescences in place of tendrils (Boss and Thomas, 2002), showing that GA inhibits flowering in grapevine. A recent work showed that in grapevine pollination and fertilization induce berry growth and trigger the expression of a *GA20ox*, whereas in unpollinated carpels the expression of *GA20ox* is induced (Dauelsberg *et al.*, 2011). So far these are the only two GA metabolism genes identified in grapevine, but no functional characterization has been provided yet.

What is known about GA metabolism in grapevine is mainly limited to automatic gene prediction and annotation (http://genomes.cribi.unipd.it/grape/, www.genoscope.cns.fr/spip/ Vitis-vinifera-e.html, and http://genomics.research.iasma.it, last accessed 23 July 2013), but their function, tissue specificity, and timing of expression have been neither verified nor explored. In this article, a comprehensive study of the family of GA oxidases in *V. vinifera* is presented, providing their *in vitro* and *in vivo* functional characterization, transcript expression, tissue localization, and evolutionary analyses. The results led to a comprehensive annotation and characterization of these proteins in grapevine, and provide a significant contribution to the understanding of the complexity of the GA regulatory pathway and the evolution of GA oxidases in plants. Furthermore, the combination of different data on GA oxidases and on the accumulation of endogenous GAs provides insights on the control of GA homeostasis in the grapevine flower and around fruit-set.

## Materials and methods

### Phylogenetic reconstructions

GA oxidase amino acid sequences of grapevine, *A. thaliana* (www.arabidopsis.org, 23 July 2013), additional sequences from *Glycine max* and *Oryza sativa* (www.phytozome.net, 23 July 2013) and according to nomenclature indicated by Han and Zhu (2011), plus a number of additional sequences of characterized GA oxidases were aligned using PRANK (Markova-Raina and Petrov, 2011) with two iterations and estimating the tree during alignment. Two types of analyses were performed: a non-parametric bootstrap analysis on 100 pesudo-replicates using RAxML (Stamatakis, 2006); and two independent Bayesian Monte Carlo Markov Chains using PhyloBayes 3 (Lartillot *et al.*, 2009), stopping the chains after the two runs had satisfactorily converged. For both analyses, the LG model (Lee and Gascuel, 2008) was used, coupled with an empirical estimation of the amino acid frequencies and four discrete categories of a gamma distribution to account for among-site rate variations. All analyses were carried out on two different types of alignment, one done using one single iteration of PRANK (www.ebi.ac.uk/goldman-srv/prank/prank/, last accessed 23 July 2013) at default settings, and one using MUSCLE (Edgar, 2004).

### Plant material

Plants of *V. vinifera* Pinot Noir, clone ENTAV115 (Velasco *et al.*, 2007) were used for cloning GA oxidase genes, and for the expression analysis in different organs. Berries were collected at three maturation stages: pea-size (BP, retaining the forming seed); green-hard (BGH, deprived of seeds); and post-véraison stage (BPV, deprived of seeds). These correspond to developmental stages EL29, EL33, and EL36-37 according to Coombe (1995). Roots were obtained from branches planted in turf. The floral organs were obtained from frozen inflorescences collected at anthesis (50% of open flowers) and separated into rachis (Ra), closed flowers (Fl), and open flowes. The latter were further separated into calyptra (Cal); stamen and pollen (S/P); and carpel (Car).

Endogenous GA measurements and the quantitative reverse transcription–PCR (qRT–PCR) experiments in inflorescences and during fruit-set were performed on material harvested from a vineyard of Pinot Gris (clone R6) in 2010. Given the high heterogeneity of these samples, three biological replicates were considered, each consisting of multiple inflorescences deprived of their rachis. Anthesis was selected as the day on which 50% of inflorescences in the vineyard were open.

For the RNA-seq experiment, inflorescences at anthesis (deprived of the rachis) were harvested from 1-year-old pot-grown Pinot Gris (clone R6), at two time points (14:00h and 18:00h) during the day at which half of their flowers were open.

### Identification of the GA oxidases and study of their gene structure

*Arabidopsis thaliana* GA oxidase protein sequences were used as the query in a BLASTp search against the *V. vinifera* gene predictions based on the 12× Pinot Noir genomes (www.genoscope.cns.fr/spip/Vitis-vinifera-e.html; http://genomes.cribi.unipd.it/grape/; http://genomics.research.iasma.it; Jaillon *et al.*, 2007; Velasco *et al.*, 2007). The full-length coding sequences were cloned in pENTR/D-topo from Pinot Noir—either from cDNA pools obtained from different tissues or from the tissue in which expression was the highest—and sequenced. The primers used are reported in Supplementary Table S2 available at *JXB* online. The coding regions of GA oxidases were deposited in GenBank with the following accession numbers: KC898178 (VIT_05s0020g01310); KC898187 (VIT_16s0098g00860); KC898176 (VvGA3ox1); KC898175 (VvGA3ox2); KC898177 (VvGA3ox3); KC898179 (VvGA2ox1); KC898180 (VvGA2ox2); KC898181 (VvGA2ox3); KC898182 (VvGA2ox4); KC898183 (VvGA2ox5); KC898184 (VvGA2ox7); KC898185 (VvGA2ox6); KC898188 (VvGA20ox1); KC898186 (VvGA20ox2); and KC898189 (VvGA20ox3) (Dauelsberg *et al.*, 2011).

### qRT–PCR expression analysis

RNA was extracted using a Spectrum Plant Total RNA kit (Sigma). cDNA was synthesized from 1.5 μg of DNase-treated total RNA using Superscript VILO (Invitrogen) according to the manufacturer’s instructions, and diluted 10- to 20-fold prior to amplification.

qRT–PCR of genes around anthesis and fruit-set was performed with an ABI-PRISM7000 (Applied Biosystems), using the instrument default protocol; whereas qRT–PCR of genes in different tissues was performed with Viia7 (Applied Biosystems) using the standard fast protocol. For normalization, two or three housekeeping genes were selected among the six most stable ones using GeNORM (Vandesompele *et al.*, 2002; Pfaffl *et al.*, 2004). Amplification efficiencies were calculated using Linreg (Ruijter *et al.*, 2009), and used to calculate normalized relative quantities (NRQs) according to Pfaffl (2001). Statistical analysis was performed with a Student’s *t*-test on log_2_(NRQ) according to Rieu and Powers (2009). Primers used for qRT–PCR analysis are reported in Supplementary Table S2 at *JXB* online.

### Extraction of endogenous GAs

Frozen inflorescences (1g) deprived of the rachis were ground and lyophilized, and were extracted twice with 5ml of 75% methanol: overnight at –20 °C and then on ice for 2h, with stirring. An aliquot of 200ng of deuterated GAs was added to the extract and passed through a Sep-Pack tC18 (6ml, Waters) and then a MCX cartridge (3ml, Waters) according to Dobrev and Kamínek (2002) and Hirano *et al.* (2007).

Eluted, dried-down fractions were suspended in 0.5ml of 50% methanol, 0.1% formic acid (FA). GA standards (GA_1_, GA_3_, GA_4_, GA_8_, GA_9_, GA_12_, GA_20_, GA_29_, GA_34_, GA_51_, GA_53_), and deuterated GAs (^2^H_2_-GA_1_, ^2^H_2_-GA_4_, ^2^H_2_-GA_9_, ^2^H_2_-GA_8_, ^2^H_2_-GA_20_, ^2^H_2_-GA_3_) were purchased from OlChemIm. Six to eight biological replicates of inflorescence pools were used.

### In vitro activity of GA oxidases

Full-length coding sequences of GA oxidases were cloned in pENTR/D-topo (Invitrogen), and recombined into pDEST15 (Invitrogen) using a Gateway LR Clonase II enzyme mix (Invitrogen). *Escherichia coli* BL21 (DE) pLYSs expressing N-terminal glutathione *S*-transferase (GST) fusion proteins were grown at 37 °C to an OD_600_ of ~0.5, and then induced for 4h at 28 °C with 1mM isopropyl-β-d-thiogalactopyranoside (IPTG). Expression was checked by SDS–PAGE after partial purification of the *E. coli* lysate on glutathione–Sepharose 4B (GE-Healthcare). The activity assay was performed on the soluble fraction of the bacterial lysate essentially as reported by Bou-Torrent *et al.* (2011). Reaction mixtures (100 μl) were incubated at 30 °C and stopped after 4h by addition of 100 μl of methanol, 0.2% FA. Substrate preferences were tested by incubating at 25 °C for either 30min or 1h.

### Identification and quantification of GAs

Separation of the GA compounds was performed on a Acquity HSS T3 column 1.8 μm, 100 mm×2.1mm (Waters) maintained at 40 °C, with mobile phases of 0.1% FA in water (A), and 0.1% FA (B) in acetonitrile, a flow rate of 0.4ml min^–1^, and injection volume of 5 μl. The gradient profile was: 0min, 5% B; (0–3min), linear gradient to 20% B; (3–4.3min), isocratic 20% B; (4.3–9min), linear gradient to 45% B; (9–13min), linear gradient to 80% B; (13–15min), 100% B; (15.01–17min), 5% B.

Quantification was performed in the multiple reaction monitoring mode in a Xevo-MS/MS (Waters) by comparison with standard curves. The transitions are reported in the Supplementary text S1 at *JXB* online.

### Overexpression of GA oxidases in Arabidopsis thaliana

pENTR/D-topo (Invitrogen) clones were recombined into pK7WG2 (Karimi *et al.*, 2002) for extopic expression. *Arabidosis thaliana* Col-0 plants were transformed by floral dipping with *Agrobacterium tumefaciens* GV3101 carrying the pK7WG2 clones, and T_1_ positive plants were selected on 0.5 MS plates (Murashige and Skoog, 1962) containing 50mg l^–1^ kanamycin and 250mg l^–1^ cefotaxime. GA_3_ at 100 μM was added to the plates for selection of T_1_ plants overexpressing *GA2ox* genes.

## Results

### GA oxidase sequence analysis and phylogenetic tree

**Fig. 2.**
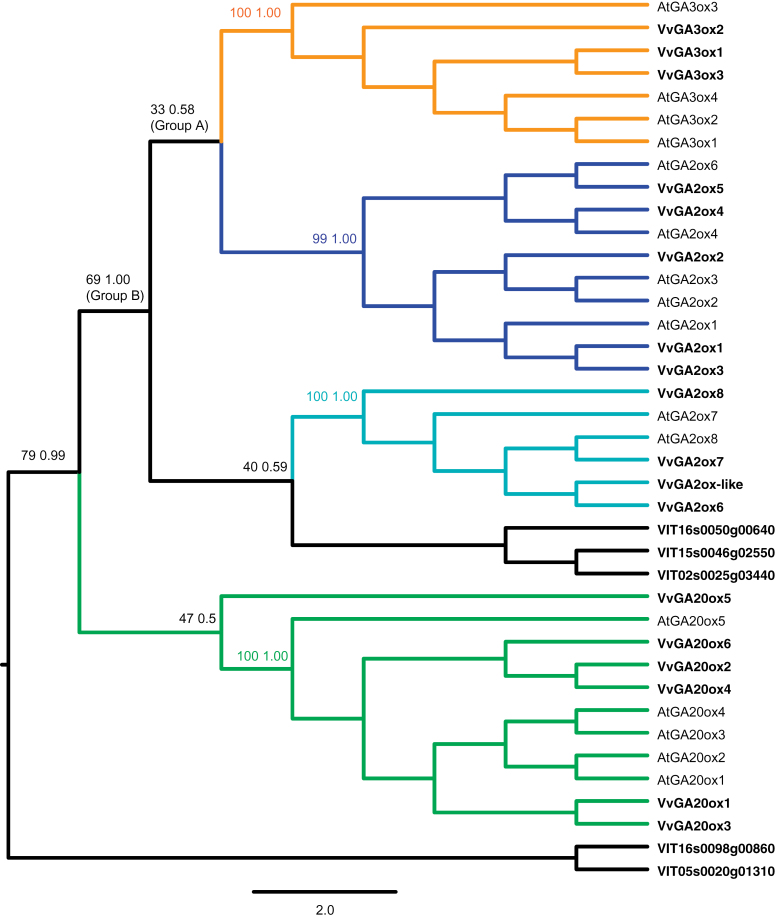
Phylogenetic tree of GA oxidases in *V. vinifera* and *A. thaliana*. The tree is the simplified version of the maximum likelihood tree of a larger alignment using more taxa (Supplementary Fig. S3 at *JXB* online). Supports at nodes are BS from the analyses of 100 pseudo-replicates (under maximum likelihood) and Bayesian PP using PhyloBayes and the LG+G model. The tree supports a sister relationship between GA3ox and C_19_-GA2ox proteins (group A). Most GA oxidases of *V. vinifera* (in bold) cluster in four clearly distinct subgroups. (This figure is available in colour at *JXB* online.)

Based on sequence similarity, 23 putative GA oxidases were initially identified in the *V. vinifera* genome. Their identifiers, accession numbers, and amino acid sequences are reported in Supplementary Table S1 at *JXB* online. These genes were validated by coupling functional experiments with the presence of known functional motifs and their phylogenetic relationships. Based on this last analysis, VIT_05s0020g01310, and VIT_16s0098g00860 appeared genetically divergent, although they possessed typical motifs of 2-ODDs (Lukačin and Britsch, 1997; Kang *et al.*, 2002). The corresponding recombinant proteins, tested in this work, did not show any GA oxidase activity, and were therefore used as the outgroup for the phylogenetic analyses. VIT_15s0046g02550, VIT_16s0050g00640, and VIT_02s0025g03440 were also slightly divergent and did not cluster with any known GA oxidase subgroup in the phylogenetic tree (Fig. 2 and below). An additional sequence (VIT_19s0177g00020) lacked one histidine of the iron-binding site, and was therefore named *GA2ox-like* and not further analysed.

Exclusion of divergent proteins led to a set of 17 putative *V. vinifera* GA oxidases: six GA20ox, three GA3ox, and eight GA2ox proteins. All these proteins possess the catalytic iron-binding triad (HDH), the R and S residues involved in the binding of 2-oxoglutarate, and the conserved leucine residue that was suggested to abolish the functionality of Os20ox2 when mutated in the rice *semi-dwarf 1* mutant (Spielmeyer *et al.*, 2002) (Supplementary Fig. S1 at *JXB* online). The motif NyYPXCXXP, considered to also be involved in the binding of 2-oxoglutarate (Xu *et al.*, 1995; Kang *et al.*, 2002), was also fairly conserved. Candidate GA20ox proteins show, in addition, conservation of the LPWKET motif which is suggested to be involved in binding the GA substrate (Xu *et al.*, 1995). An attempt was made to determine motifs which may be associated with the specific GA oxidase function as in Han and Zhu (2011). Overall it was found that such motifs (HYRADMNTLDAFTNW and QPHIPMQFIWPDEEK) are more conserved in *VvGA20ox3* and *VvGA20ox1* than in *VvGA20ox2*, *VvGA20ox4*, *VvGA20ox5*, and *VvGA20ox6* (Supplementary Fig. S1)

In order to determine whether they encode functional GA oxidases and to help their proper annotation, the coding sequences of 13 of the identified genes were cloned. Sequencing of their full-length cDNA allowed curation of existing gene predictions. The gene structure is conserved within each functional class: bona fide *GA3ox* genes display two exons, whereas *GA20ox* and *GA2ox* genes display a three-exon gene structure, with the exception of *VvGA20ox5* whose structure is based on prediction (Supplementary Fig. S2 at *JXB* online). Despite several attempts with different primer pairs, the full-length sequences of *VvGA20ox4*, *VvGA20ox5*, *VvGA20ox6*, and *VvGA2ox8* could not be amplified from Pinot Noir; thus it was not possible to study these genes further.

In order to assess their ontology and to obtain a clearer picture of their evolution, a phylogenetic tree of *V. vinifera* GA oxidases was generated adding orthologues of characterized GA oxidases from various species. According to the maximum likelihood and Bayesian trees (Fig. 2; Supplementary Fig. S3 at *JXB* online), most of the putative grapevine GA oxidases cluster in the previously described functional groups: GA20ox, GA3ox, and GA2ox proteins. The latter group is further di vided into two clearly distinct, non-monophyletic functional classes: C_19_-GA2ox proteins, including VvGA2ox1–VvGa2ox5, and the C_20_-GA2ox class including VvGA2ox6–VvGA2ox8, the latter characterized by the presence of the unique amino acid sequence (SYRWGxPSATSxxQxSxSEAFH) described in their *Arabidopsis* orthologs by Schomburg *et al.* (2003).

The homology of *VvGA20ox5*, VIT_16S0050g00640, VIT_02s0025g03440, and VIT_15s0046g02550 is less clear, and it will be further addressed in the Discussion.

### Grapevine GA oxidases display substrate preference in vitro

Grapevine GA oxidases were expressed as recombinant GST fusion proteins in *E. coli*. Successful expression (VvGA2ox2, VvGA2ox3, VvGA2ox4, VvGA2ox5, VvGA2ox7, VvGA3ox1, VvGA3ox2, VvGA3ox3, VvGA20ox1, VvGA20ox2, and VvGA20ox3) was verified by SDS–PAGE of the soluble *E. coli* lysate after purification on glutathione beads (not shown). Cell cultures induced for expression were used for activity tests according to Bou-Torrent *et al.* (2011). In each test, the presence/absence of GA_1_, GA_4_, GA_8_, GA_34_, GA_20_, GA_9_, GA_51_, GA_29,_ GA_12_, and GA_53_ was determined by UPLC-MS/MS (ultra performance liquid chromatography–tandem mass spectrometry), and the results are shown in Table 1.

**Table 1.** In vitro *activity of recombinant grapevine GA oxidases* *Escherichia coli* lysates expressing recombinant GA oxidases (column 1) were incubated with a GA substrate (column 2) for 4h at 30 °C. Each recombinant protein was tested at least three times with each of the following precursors: GA_1_, GA_4_, GA_20_, GA_9_, GA_12_, and GA_53_. The following GAs were detected in the reaction by UPLC-MS/MS: GA_1_, GA_4_, GA_8_, GA_9_, GA_20_, GA_34_, GA_29_, GA_51_, GA_12_, and GA_53_. The observed product is reported in column 3, and the corresponding activity in column 4. Recombinant GA2ox proteins were also tested for GA20ox activity with GA_12_ and GA_53_, and no product among those indicated was detected in multiple experiments.

Protein	Substrate	Products	Activity
GST-VvGA20ox1	GA_12_	GA_9_	GA20ox
	GA_53_	GA_20_	GA20ox
GST-VvGA20ox2	GA_12_	GA_9_	GA20ox
	GA_53_	None	
GST-VvGA20ox3	GA_12_	GA_9_	GA20ox
	GA_53_	GA_20_	GA20ox
GST-VvGA3ox1	GA_9_	GA_4_	GA3ox
	GA_20_	GA_1_	GA3ox
	GA_4_	—	GA3ox
	GA_1_	—	
GST-VvGA3ox2	GA_9_	GA_4_	GA3ox
	GA_20_	GA_1_	GA3ox
	GA_4_	—	
	GA_1_	—	
GST-VvGA3ox3	GA_9_	GA_4_	GA3ox
	GA_20_	GA_1_	GA3ox
	GA_4_	—	
	GA_1_	—	
VvGA2ox2	GA_9_	GA_51_	GA2ox
	GA_20_	GA_29_	GA2ox
	GA_4_	GA_34_	GA2ox
	GA_1_	GA_8_	GA2ox
VvGA2ox3	GA_9_	—	
	GA_20_	—	
	GA_4_	GA_34_	GA2ox
	GA_1_	GA_8_	GA2ox
VvGA2ox4	GA_9_	GA_51_	GA2ox
	GA_20_	GA_29_	GA2ox
	GA_4_	GA_34_	GA2ox
	GA_1_	GA_8_	GA2ox
GST-VvGA2ox5	GA_9_	GA_51_	GA2ox
	GA_20_	GA_29_	GA2ox
	GA_4_	GA_34_	GA2ox
	GA_1_	GA_8_	GA2ox
GST-VvGA2ox7	GA_9_	GA_51_	GA 2ox
	GA_20_	—	
	GA_4_	GA_34_	GA2ox
	GA_1_	GA_8_	GA2ox

Recombinant VvGA2ox2, VvGA2ox4, and VvGA2ox5 showed the expected GA2ox activity on both 13-hydroxylated and non-13-hydroxylated substrates. VvGA2ox3 displayed limited activity and only with GA_1_ and GA_4_ as substrates, but not their C_19_-GA precursors (GA_20_ and GA_9_), probably due to low expression and low protein activity. Although it was not possible to produce recombinant VvGA2ox1, its function is presumed to be similar to that of VvGA2ox3, based on their high amino acid identity.

Based on its similarity to C_20_-GA2ox proteins, VvGA2ox7 was expected to act exclusively on C_20_-GA substrates like its *A. thaliana* orthologue (Schomburg *et al.*, 2003). However, the activity assays showed that the recombinant protein was active on C_19_-GAs such as GA_4_, GA_1_, and GA_9._ Activity on C_20_-GAs was not tested (Table 1).

When recombinant VvGA3ox1 was incubated with GA_9_, both 3β-hydroxylation products (GA_4_) and occasionally 2β-hydroxylation products were detected (GA_34_ and GA_51_). On the other hand, VvGA3ox2 and VvGA3ox3 showed only GA3ox activity.

VvGA20ox1, VvGA20ox2, and VvGA20ox3 all displayed GA20ox activities: VvGA20ox1 and VvGA20ox3 were capable of using both GA_12_ and GA_53_ as substrates, whereas VvGA20ox2 was active on GA_12_, but never on GA_53_ in several repeated experiments.

In order to understand the diversity of the family in grapevine, proteins with a lower degree of conservation (VIT_05s0020g01310 and VIT_16s0098g00860) were also included the analysis. Neither of them showed any GA oxidase activity, and thus served as negative controls in the tests, and as the outgroup in the phylogenetic analyses.

**Fig. 3.**
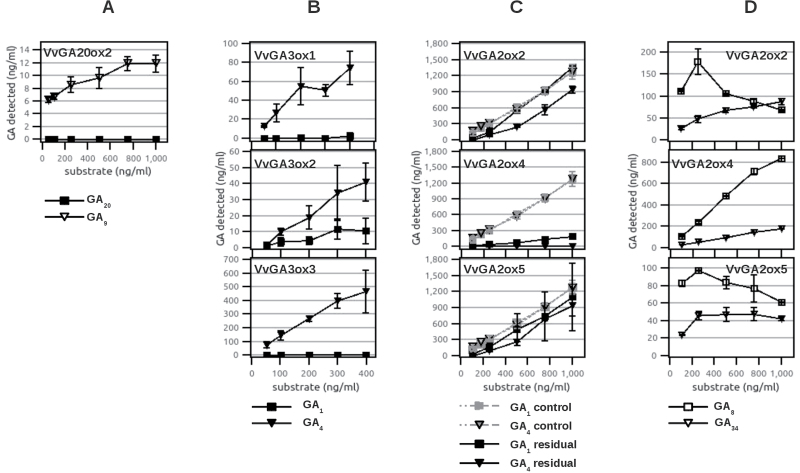
Substrate preference of grapevine GA oxidases *in vitro*. Substrate preferences of *E. coli* lysate expressing recombinant GA oxidases when supplied with increasing concentrations of substrate mixture; on the *x*-axis is reported the concentration of substrates supplied to the reaction, whereas on the *y*-axis is the concentration of residual substrates and/or products formed. Error bars show the SD. (A) Activity of recombinant VvGA20ox2 supplied with increasing concentrations of GA_12_ and GA_53_ produces GA_9_ (triangles) but not GA_20_ (filled squares); *n*=4. (B) Activity of GA3ox proteins: recombinant VvGA3ox1, VvGA3ox2, and VvGA3ox3 supplied with increasing concentrations of GA_9_ and GA_20_ produce GA_4_ (filled triangles) and GA_1_ (filled squares); *n*=3. (C and D) Activity of recombinant VvGA2ox1, VvGA2ox4, and VvGA2ox5 when supplied with increasing concentrations of GA_1_ and GA_4_. (C) Residual substrates detected in the reactions are represented by black squares (GA_1_) and black triangles (GA_4_) (*n*=2), and controls are represented by grey squares (GA_1_) and triangles (GA_4_) (*n*=5). Controls are the substrates measured in parallel experiments conducted with *E. coli* lysates expressing proteins with no GA oxidase activity (VIT_16s0098g00860) and take into account systematic errors and ion suppressions, allowing the substrate consumption due to protein activity to be determined effectively. (D) Deactivation products detected in the reactions are represented by open squares (GA_8_) and triangles (GA_34_) (*n*=2)

To assess substrate preference, cultures expressing the recombinant enzymes were tested with increasing concentrations of mixtures of 13-hydroxylated and non-13-hydroxylated substrates, and both the residual substrates and the products were measured. These results are reported in Fig. 3. Again, VvGA20ox2 was never active on GA_53_ when provided in a mixture with GA_12_ in many repeated experiments (Fig. 3A). Recombinant VvGA20ox1 and VvGA20ox3 could both convert GA_12_ and GA_53_ into their respective C_19_ products, but low and scarcely reproducible activities did not allow conclusions to be drawn on their possible substrate preference. Similarly, all three GA3ox proteins preferred non-13-hydroxylated substrates, and VvGA3ox3 appeared incapable of using GA_20_ at all, since GA_1_ was not detected (Fig. 3B).

Recombinant GA2ox proteins tested with mixtures of GA_1_ and GA_4_ showed production of both GA_8_ and GA_34_ (Fig. 3C, D). Since the GA_8_-catabolite and GA_34_-catabolite standards—which are possible end-products of GA2ox reactions—were not available, substrate preference of GA2ox proteins were tested by measuring the concentrations of substrates remaining after the reaction, and by comparing them with those added to controls, consisting of *E. coli* cultures expressing control proteins (Fig. 3C). Recombinant VvGA2ox2 displayed limited activity, and a slight preference for GA_4_ over GA_1_ only at elevated substrate concentrations. Recombinant VvGA2ox4 was very active, and preferred GA_4_ over GA_1_ at all concentrations tested: GA_4_ was completely converted in all trials, whereas some residual GA_1_ was always detected. The low levels of GA_34_ detected may indicate a rapid further oxidation (e.g. conversion into GA_34_-catabolite). VvGA2ox5 displayed limited activity, but no significant substrate preference in the present experimental conditions.

### VvGA 2-oxidases are functional in vivo

The coding sequences of grapevine *VvGA2ox2*, *VvGA2ox3*, *VvGA2ox5*, and *VvGA2ox7* were ectopically expressed in *A. thaliana* wild-type plants (Col-0) to confirm their function *in vivo*. Multiple independent lines were obtained for each construct, with different levels of expression of the transgene, as determined by qRT–PCR (not shown). Plants overexpressing *VvGA2ox2*, *VvGA2ox3*, *VvGA2ox5*, and *VvGA2ox7* were severely dwarf as compared with the wild type (Supplementary Fig. S4 at *JXB* online), with reduced rosette diameter, shorter internodes, and darker and epinastic leaves. Flowering was considerably delayed in all lines, and transgenic plants showed the typical bushy phenotype of GA-deficient mutants (Coles *et al.*, 1999; Schomburg *et al.*, 2003), with shorter siliques and different degrees of infertility, rescued by applications of GA_3_. These data confirmed the *in vitro* activity results for VvGA2ox2, VvGA2ox3, and VvGA2ox5, and provided indications that VvGA2ox7 (a putative C_20_-GA2ox) is also functional.

### Tissue specificity of grapevine GA oxidases

**Fig. 4.**
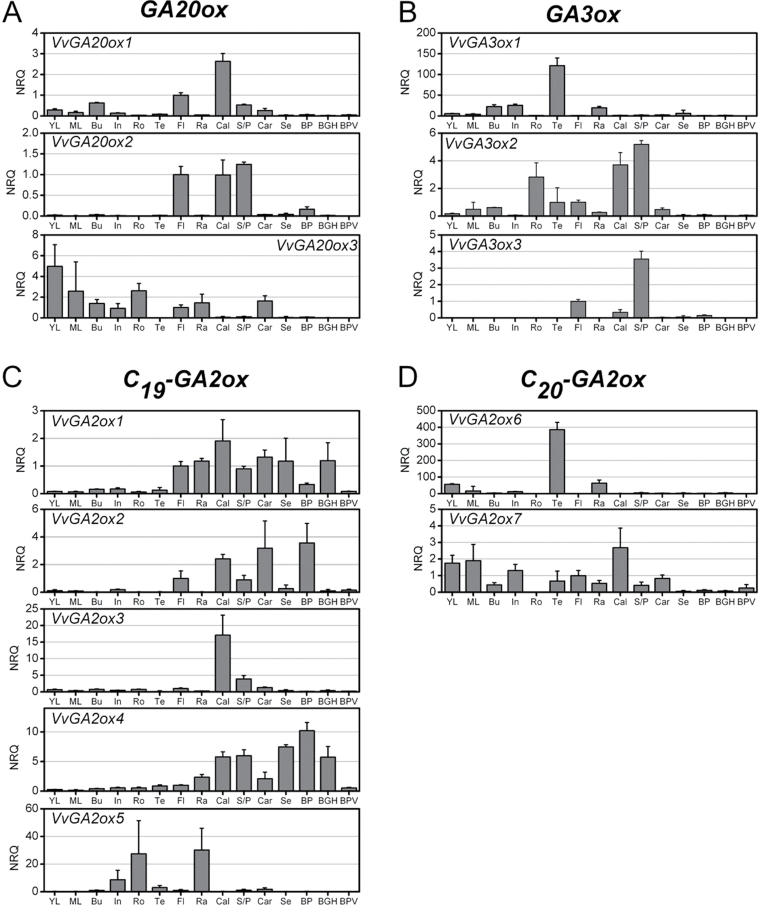
Relative quantification of GA oxidase transcripts in different *V. vinifera* organs as determined by qRT–PCR. Relative expression of GA oxidase genes whose full-length coding sequence could be cloned grouped according to their functional class (experimentally confirmed or predicted): *GA20ox* (A), *GA3ox* (B) *C*_*19*_ *-GA2ox* (C), and (predicted) *C*_*20*_ *-GA2ox* (D). Vertical bars represent the normalized relative quantity (NRQ) of GA oxidase transcripts in three biological replicates (error bars indicate the SD, *n*=3). Normalization was performed against the relative quantities of *ACTIN* and *SAND*. The analysed organs were: young leaf (YL); mature fully expanded leaf (ML); green bud (Bu); green internode (In); root (Ro); and tendril (Te). The floral organs were obtained from inflorescences at anthesis (50% of flowers retaining their calyptra) and were: whole unopened flower (Fl); rachis (Ra); detached calyptra (Cal); stamen and pollen of open flowers (S/P); and carpel (Car) of open flowers. The berry organs were: seed (Se) from berries at the green-hard stage; whole berry at the pea-size stage (BP; stage EL29 according to Coombe, 1995); berry (deprived of seeds) at the green-hard stage (BGH, stage EL33), and, finally, berry (no seeds) at post-véraison (BPV, stage EL36–37). NRQ of the unopened flower is normalized to 1.

In order to understand the role of the different GA oxidases in the maintenance of the active GA pool in *V. vinifera*, their relative expression was analysed by qRT–PCR in different organs: young and mature (fully expanded) leaves, roots, green buds, green seeds, berries at three different stages, internodes, and tendrils, and different parts of the inflorescence at anthesis. The results are shown in Fig. 4.

Several genes are predominantly expressed only in a subset of organs, mainly the flower, whereas expression in the fruit is restricted to stages preceding the véraison of berries.

*VvGA20ox1* and *VvGA20ox2* are mainly expressed in flowers, predominantly in calyptras and stamen/pollen, but their relative expression remains low in the carpel (Fig. 4A). On the other hand, expression in the flower of *VvGA20ox3* (Fig. 4A) seems restricted to the carpel. *VvGA3ox3* is virtually specific to stamen/pollen, whereas *VvGA3ox2* transcript is abundant in the male organs, calyptras, and roots, although it is also detected in the carpel, bud, and leaves (Fig. 4B).

With the exception of *VvGA2ox6*, all other *GA2ox* transcripts are detected in the flower, but are differentially expressed in different floral organs: *VvGA2ox1* is found in all flower parts, whereas *VvGA2ox2* is missing in the rachis; *VvGA2ox3* is virtually flower specific and abundant in calyptras, whereas *VvGA2ox5* is almost exclusively expressed in the rachis, in roots and internodes (Fig. 4C).

*VvGA3ox1* and *VvGA2ox6* transcripts accumulate mostly in tendrils (Fig. 4B, D), whereas *VvGA2ox7* seems to be ubiquitously expressed (Fig. 4D). *VvGA2ox4*, although present in all floral organs, shows higher expression in seeds and fruits prior to véraison (Fig. 4C).

### Absolute quantification of GA oxidase transcripts in grapevine inflorescences

**Fig. 5.**
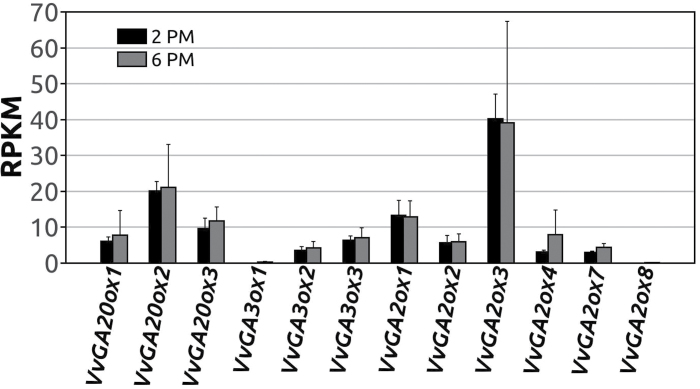
RNA-seq expression data of GA oxidases in inflorescences of Pinot Gris. The plot reports average expression values (RPKM) of grapevine GA oxidases in inflorescences of Pinot Gris at anthesis, harvested at two time ponts during the day (14:00h, black bars; and 16:00h, grey bars). Error bars indicate the SD, *n*=3.

The qRT–PCR analysis showed a high degree of compartmentalization of the expression of GA oxidase isoforms; however, being a relative quantification, it did not allow the determination of which of the identified genes may be driving the accumulation of bioactive GAs in flowers. For this reason, RPKM (reads per kilobase per million mapped reads) (Mortazavi *et al.*, 2008) values of grapevine GA oxidase genes were extracted from an RNA-seq data set of inflorescences of Pinot Gris harvested at anthesis (Fig. 5). Over 287 million Illumina sequences were matched against a database of Pinot Noir gene predictions (annotation V1). Sequences were also aligned with the corresponding genomic regions of predicted GA oxidase genes to check that low or null RPKM values were not simply due to incorrect gene predictions. No significant matches were found when aligning the sequences against the genomic regions of *VvGA2ox6*, *VvGA20ox4*, *VvGA20ox5*, *VvGA20ox6*, or *GA2ox-like*, supporting the hypothesis that these genes are not expressed in inflorescences. The expression of *VvGA2ox8* and *VvGA3ox1* was also insignificant (<0.2 RPKM), while the expression of the remaining GA oxidase genes ranged from 0.5 to 40 RPKM.

In flowers, the absolute expression of *VvGA20ox2* is almost 2-fold as compared with that of *VvGA20ox3*, and 4-fold compared with *VvGA20ox1.* The expression of *VvGA3ox3* is at least 1.5-fold as compared with that of *VvGA3ox2*, whereas the *VvGA3ox1* transcript is at least 20- to 80-fold less abundant. Among the *GA2ox* genes, *VvGA2ox3* is the predominant transcript, and only *VvGA2ox7* is expressed among the putative *C__*20*__-GA2ox* genes.

### Quantification of GAs during fruit-set

**Fig. 6.**
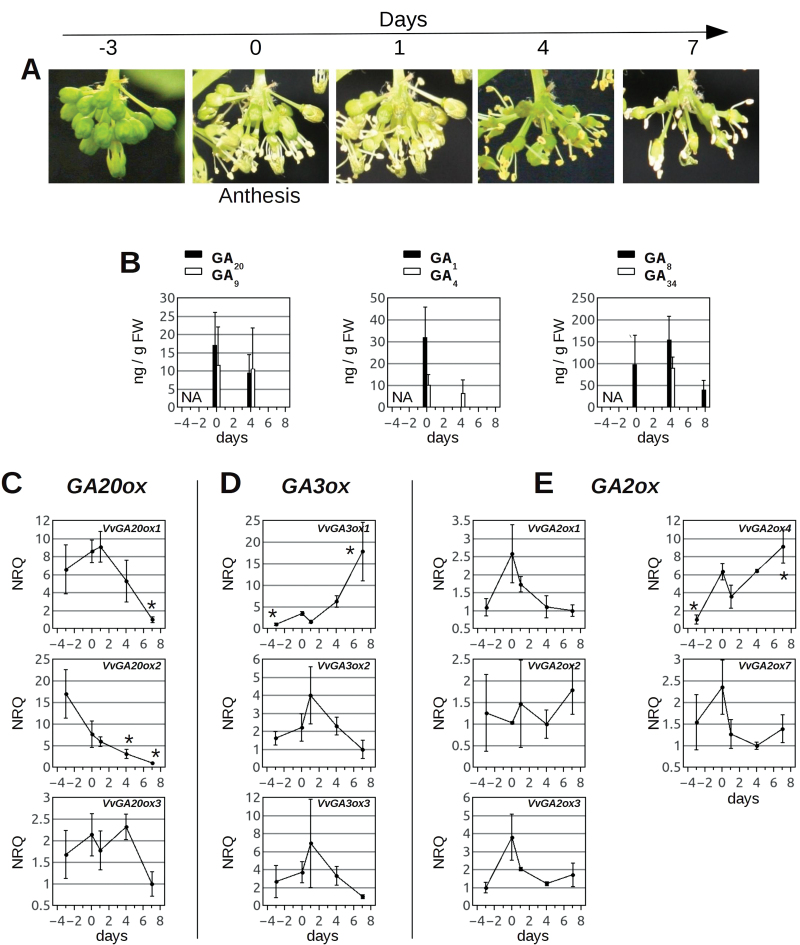
Accumulation of endogenous GAs and GA oxidase transcripts in inflorescences of Pinot Gris during fruit-set. (A) Section of an inflorescence of Pinot Gris in a time window of 3 d prior to anthesis until 7 d after anthesis. (B) Quantification of endogenous GAs in inflorescences of Pinot Gris at anthesis (0) and after 4 d and 8 d. At time point 0, ~50% of the flowers on the inflorescence were open, whereas ~50% retained their calyptra. After 8 d, some carpels were enlarging into small fruits. Vertical bars indicate the average quantity of GAs in ng g^–1^ of frozen weight; error bars indicate the SD. Data were used for the average only where GAs were detected in at least half of the biological replicates (6–8) analysed. 13-Hydroxylated GAs are represented by filled bars (GA_1_, GA_20_, GA_8_) and non-13-hydroxylated compounds are represented by open bars (GA_4_, GA_9_, GA_34_); NA, not analysed. (C–E) Averaged NRQs of grapevine *GA20ox* (C), *GA3ox* (D), and *GA2ox* (E) genes expressed in inflorescences of Pinot Gris, as measured by qRT–PCR, at anthesis (time point 0), 3 d prior to anthesis (–3), and after 1, 4, and 7 d. Relative quantities are normalized against the expression of *ACTIN*, *GADPH*, and *EF1α*. Asterisks indicate transcript accumulations that are significantly different (*P* < 0.05) from the corresponding value at anthesis. (This figure is available in colour at *JXB* online.)

In order to understand GA metabolism during anthesis and fruit-set, the content of bioactive GAs, their precursors, and deactivation products was measured in inflorescences and fruitlets of Pinot Gris. Anthesis is hereby considered as the window in which ~50% of the flowers are closed and retain their calyptra. At 8 d following anthesis, most flowers have lost their calyptra and most stamens, and carpels have started enlarging (Fig. 6A). GAs were extracted at anthesis (time point ‘0’), and 1, 4, and 8 d later. The plant material was analysed for its content of GA_1_, GA_3_, GA_4_, GA_9_, GA_20_, GA_34_, GA_8_, GA_29_, and GA_51_. Grapevine inflorescences proved a difficult material to work with, due to the amount of the quantified GA species often being close to their detection limit (e.g. GA_51_ was never detected) and to interfering substances. Reliable quantifications were obtained of GA_1_ and GA_4_, and their direct deactivation products (GA_8_ and GA_34_), and precursors (GA_20_ and GA_9_). At 1 d after anthesis, flowers undergo rapid developmental changes and are asynchronous on the inflorescence. Measurements at this time point showed extremely high variability and are therefore not shown here. GA_1_ and GA_4_ were both detected in grapevine inflorescences: GA_1_ was predominant over GA_4_ at anthesis, and both active GAs decreased during the transition to fruit—the GA_1_ concentration decreased more rapidly than that of GA_4_—until virtually no active GAs were detected at 8 d after anthesis (Fig. 6B). Accumulation of the precursors GA_20_ and GA_9_ also decreased after anthesis, with no GA_9_ or GA_20_ detectable after 8 d, whereas GA_8_ and GA_34_ accumulation succeeded that of GA_1_ and GA_4_, peaking at 4 d after anthesis, and diminishing later on (Fig. 6B).

### Expression of GA oxidase transcripts during fruit-set

In an attempt to explain the accumulation profile of GA_1_ and GA_4_ during anthesis and fruit-set, it was decided to investigate further the expression of GA oxidases during this process. Inflorescences of Pinot Gris were analysed at 3 d prior to anthesis, at anthesis, and at 1, 4, and 7 d after. The results of this analysis are reported in Fig. 6C–E.

The expression of all *GA20ox* genes decreased from anthesis to 7 d after anthesis (Fig. 6C), and the reduction was significant for *VvGA20ox1* and *VvGA20ox2* (*P* < 0.05). The predominant inflorescence *GA3ox* genes, *VvGA3ox2* and *VvGA3ox3*, peaked at 1 d after anthesis, and then decreased (Fig. 6D). In contrast, the *VvGA3ox1* transcript, much less abundant than that of *VvGA3ox2* and *VvGA3ox3*, was significantly up-regulated at fruit-set (Fig. 6D). Most *GA2ox* transcripts displayed a common trend, peaking at anthesis and then decreasing, whereas *VvGA2ox2* expression did not show a significant change in the developmental window analysed (Fig. 6E). *VvGA2ox4*, which is also a relatively abundant transcript, peaked at anthesis, and was the only significantly up-regulated *GA2ox* transcript in the setting fruit (Fig. 6E).

## Discussion

### The complexity of the GA oxidase family in grapevine

GA oxidases have been extensively characterized in model as well as crop species and are typically encoded by small multigene families. Several GA oxidases have also been identified in tree species such as poplar (Busov *et al.*, 2003), aspen (Israelsson *et al.*, 2005; Mauriat and Moritz, 2009), and plum (El-Sharkawy *et al.*, 2012). A recent study in apple identified a *GA20ox*, *GA3ox*, and *GA2ox*, and showed that these genes are also part of small gene families (Zhao, *et al.* 2009). However, comprehensive studies on the whole GA oxidase gene family of trees are still missing, and the present study in grapevine represents the first characterization of this sort. The results led to the discovery of six *GA20ox*, three *GA3ox*, and eight *GA2ox* genes, showing a complexity of the gene family similar to that of *Arabidopsis*, rice, and soybean (Han and Zhu, 2011).

### A new hypothesis of the evolution of GA oxidases?

**Fig 7.**
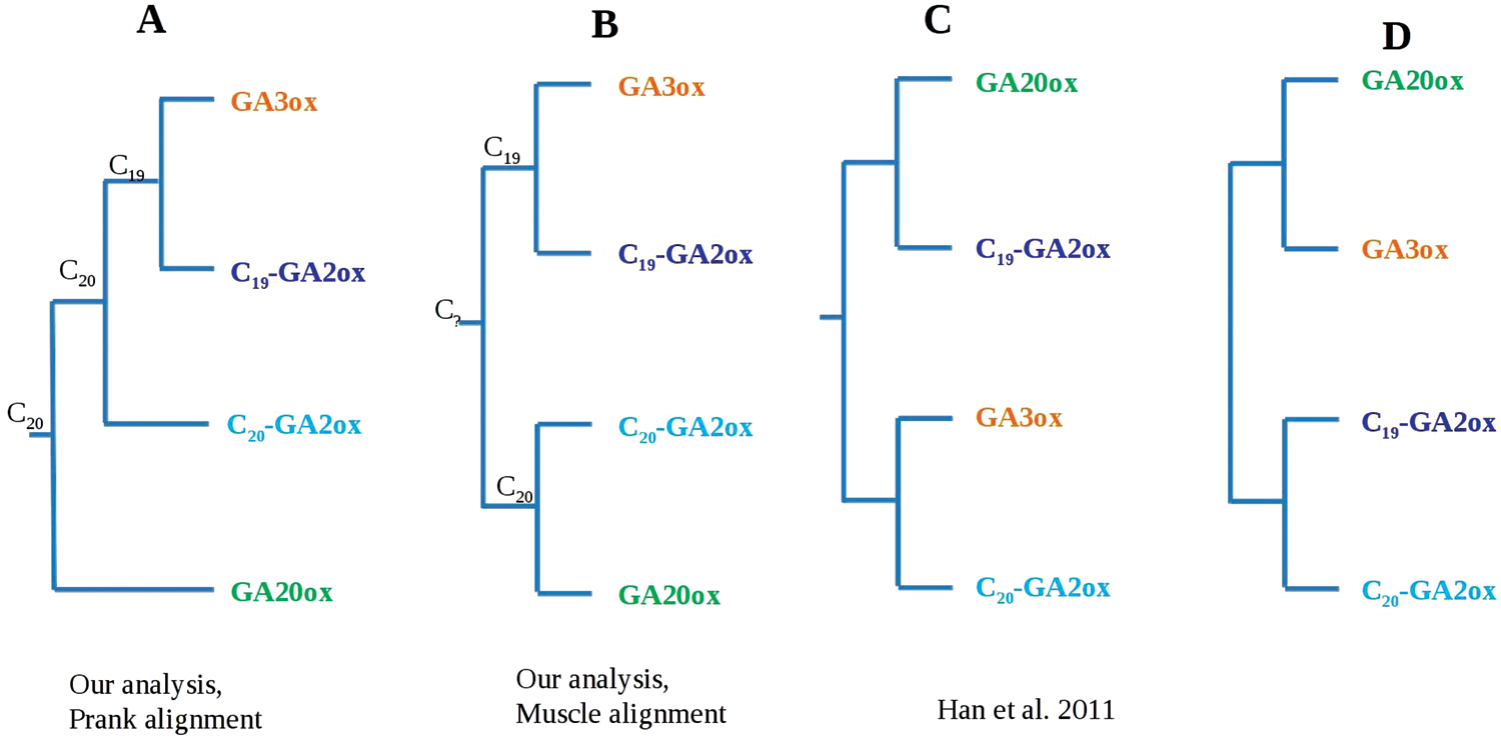
Four hypotheses for evolution of GA oxidases. The analyses (A and B) performed with either PRANK or MUSCLE alignment refer to the trees of Supplementary Fig. S3A and B at *JXB* online, respectively. Neither analysis confirms the monophyly of GA2ox proteins (C and D). The difference between hypotheses A and B relies only on the different position of the root. Both hypotheses support the C_19_-GA2ox as sister of GA3ox, suggesting a unique origin of C_19_ metabolism. (This figure is available in colour at *JXB* online.)

The phylogenetic analyses suggest a new hypothesis for evolution of GA oxidases. In the tree used here, the C_19_-GA2ox proteins are closely related to the GA3ox subgroup in what has been named group A. Although group A is the favoured topology in both a maximum likelihood (Fig. 2; and Supplementary S3A at *JXB* online) and a Bayesian analysis (Supplementary Fig. S3B), the corresponding phylogenetic signal is weak and probably confined to a few sites of the alignment (bootstrap support, BS 33; posterior probability, PP 0.59). On the other hand, group A is recovered regardless of the alignment method used. There is a possible explanation for low support at some nodes: GA oxidases probably evolved by gene duplication and subsequent adaptative neo-functionalization: this may have concentrated the phylogenetic signal into only a handful of sites. The two constituent subgroups (C_19_-GA2ox and GA3ox) of group A share a common ancestor. Since both GA3ox and C_19_-GA2ox metabolize C_19_-GA substrates, it is likely that their common ancestor was also a C_19_-enzyme (see also Fig. 7A, B).

In the phylogeny of Fig. 2, and Supplementary S3A at *JXB* online, group A is sister to C_20_-GA2ox plus some other not well identified GA oxidase-like proteins (this putative clade was named group B; BS 69, PP 1.00). Group B is well supported in Supplementary Fig. S3A, but it is not recovered in all the analyses, which has been found to be dependent on the alignment algorithm used. The phylogeny of Fig. 2 and Supplementary S3A (supporting group B) is based on an alignment carried out using PRANK, a method which has been shown to ameliorate evolutionary studies (Markova-Raina and Petrov, 2011). When using putatively less well performing alignment methods, such as MUSCLE (Edgar, 2004), a different tree topology is obtained, where group A is sister to a group of C_20_-GA2ox plus GA20ox proteins (Supplementary Fig. S3B). Notably, the difference between the two analyses relies only on the position of the root. Assuming that the correct topology is that of Supplementary Fig. S3A, the most parsimonious evolutionary explanation in terms of neo-functionalization is that GA3ox proteins evolved from a GA2ox-type ancestor. More generally, according to the hypothesis of Supplementary Fig. S3A, it is possible that the ability to metabolize C_19_-GAs evolved after the ability to metabolize C_20_-GAs. This finding may have implications for understanding the evolution of GA oxidases and of the GA metabolic pathway in plants, as it suggests that the primary (more ancient) pathway involves C_20_-GAs.

The phylogenetic analyses reported here are partially dependent on the position of the root. For this reason, the results are discussed in the light of two additional hypotheses (summarized in Fig. 7). The trees (Fig. 7A, B) challenge the recent comprehensive analysis of Han and Zhu (2011), which supports a sister relationship between GA3ox and C_20_- GA2ox proteins (Fig. 7C). A proper comparison between these results and those of the present study is complicated by their tree not showing statistical support at nodes. However, the present phylogeny makes use of a larger taxon sampling and, more importantly, employs a more accurate replacement model (LG, which assigns a different replacement probability to different amino acid substitutions) than the flat Poisson distribution used by Han and Zhu (2011), which assumes all amino acid replacements to occur with the same probability. An additional hypothesis (Fig. 7D) would support a common orgin of the GA2ox proteins, in agreement with their common 2β-hydroxylase activity. This is consistent with Serrani *et al.* (2007), who claimed that C_19_- and C_20_-GA2ox proteins originated by a duplication event from a common GA2ox ancestor. Their analyses, however, were based only on GA2ox proteins, but their origin should be addressed in the presence of other GA oxidases subgroups.

The present comparison suggests that previous hypotheses may have resulted from employing less sophisticated methodological approaches. Regardless of their relative position, there is consistent evidence that the GA2ox proteins are not monophyletic [the present results in this sense are in accordance with those of Han and Zhu (2011)]. Overall, although discussion of the results points toward a common origin of the C_19_-GA oxidases, given an obvious problem of fast evolving orthologues (GA oxidases that do not clearly cluster in Fig. 2), a generally low BS, and a rooting issue, some caution is advocated in the interpretation of the present results.

Four putative GA oxidases of *V. vinifera* do not cluster within the four subgroups. Three of them form a monophyletic group (Fig. 2) and are weakly related (BS 40) but genetically distant from the GA2ox proteins, and thus deserve future investigations. Finally, VvGA20ox5 is weakly (BS 47) supported as sister to all other GA20ox proteins, and therefore is closely related, but not part of the GA20ox subgroup. It is suggested that this gene may have originated either from a GA20ox by secondary divergence or from fusion of two other GA oxidases. Since its expression was not detected in any tissue, *VvGA20ox5* may be a not functional gene.

### GA_1_ is the predominant bioactive GA in opening flowers, whereas GA_4_ is predominant at later stages

Previous reports showed that grapevine accumulates higher concentrations of GA_1_ than GA_4_ in leaves, and lower concentrations of GA_1_ than GA_4_ in internodes, although the statistical significance of these measurements is not clear (Boss *et al.*, 2003). The present data showed a higher accumulation of GA_1_ than GA_4_ in inflorescences at anthesis, whereas only GA_4_ was detected at later stages. Although multiple bioactive GA molecules are usually simultaneously detected in plants, often developmental processes are regulated by a predominant GA species. It is widely accepted that GA_4_ is the main bioactive GA in *Arabidopsis*, since its concentration is higher than that of GA_1_ and the plant is more responsive to GA_4_ as compared with GA_1_ (Talon *et al.*, 1990; Eriksson *et al.*, 2006). It should be stressed that different developmental processes may involve specific active GAs, thus a deeper understanding is essential when considering treatment of crops. In fact, even if GA_1_ is the predominant active GA in rice, anthers accumulate GA_4_ (Hirano *et al.*, 2008). Magome *et al.* (2013) speculated that GAs with different (weak or strong) activities may be advantageous to regulate different growth requirements. The control of which bioactive GA is produced in which tissue may be determined either by the tuning on the biosynthetic pathway exerted by GA 13-hydroxylase (GA13ox)—which so far have been identified in rice but not in grapevine (Magome *et al.*, 2013)—and/or by GA oxidase substrate preferences. GA is sensed by the GA receptor GID1, encoded by a unique gene in rice and three genes in *Arabidopsis*. The GID1 receptors of both species display a higher affinity for GA_4_ than GA_1_ *in vitro* (Nakajima *et al.*, 2006; Ueguchi-Tanaka *et al.*, 2007), although these two species prefer different active GAs. In grapevine, two GID1 orthologues were identified (not shown), but their affinity for GA_1_ and GA_4_ has not been tested.

The present activity assays indicated a general preference of the grapevine biosynthetic GA oxidases for non-13-hydroxylated substrates. The specific transcript localization of different GA oxidase isoforms (e.g. within the inflorescence *VvGA20ox3* is mainly expressed in the carpel), together with eventual substrate preference, may contribute to regulate GA_1_ versus GA_4_ ratios in specific tissues and developmental stages. However, previous studies on recombinant GA oxidases also showed a preference for non-13-hydroxylated GAs even when isolated from taxa where GA_1_ is predominant (Williams *et al.*, 1998; Israelsson *et al.*, 2004; Appleford *et al.*, 2006). This needs further evaluation also in light of the function of GA13ox proteins, whose encoding genes have not been determined in grapevine.

### GA metabolism in flower organs is controlled by compartmentalization and timely expression of GA metabolic genes

**Fig. 8.**
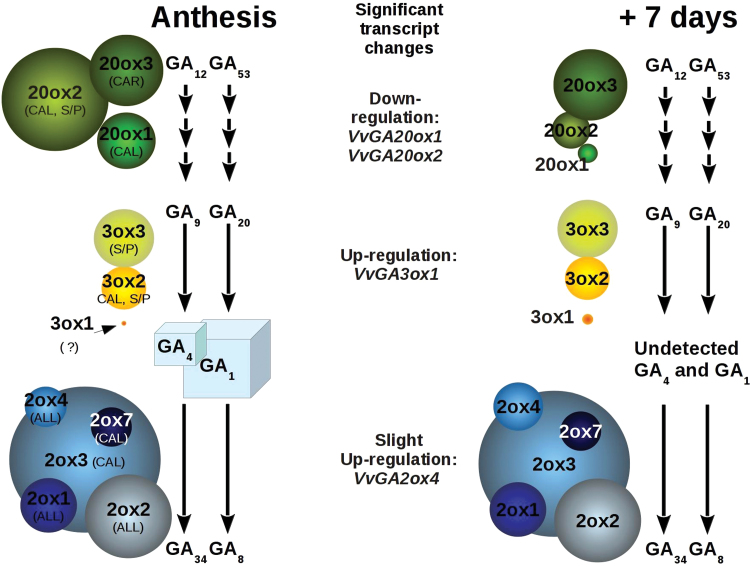
Schematic representation of GA metabolism in grapevine inflorescence. Schematic overview of GA_1_ and GA_4_ metabolism in inflorescences at anthesis (left) and after 7 d (right side). Squares represent detected GA_1_ and GA_4_, and their dimension is proportional to their concentrations. Circles represent genes whose transcripts were detected (full-length cDNA sequences) in inflorescences. Their dimension at anthesis is proportional to their transcript abundance, as determined by RNA-seq; whereas at 7 d it is proportional to their expression level as extrapolated by qRT–PCR data (only significant changes are reported, *P* < 0.05). For each gene, the flower organ in which it is predominant is indicated: CAL, calyptra; S/P, stamen and/or pollen; CAR, carpel; ALL, the transcript is distributed in all flower organs. (This figure is available in colour at *JXB* online.)

The specific localization of GA oxidase transcripts in different flower compartments suggests that they may have specific biological functions. Taken together, the qRT–PCR and RNA-seq data indicate that *VvGA20ox2* is the predominant *GA20ox* transcript in inflorescences at anthesis, suggesting an important role for this gene in driving GA synthesis during bloom (Fig. 8). Accumulation of GA at anthesis is probably a consequence of accumulation of biosynthetic enzymes in the closed flowers, as *VvGA20ox2* expression is peaking prior to bloom (Fig. 6C).

The decrease in GA_1_ and GA_4_ after anthesis (Fig. 6B) is explained by two aspects: a regulatory cue and a mechanical one. The first consists of the down-regulation of the predominant *GA20ox* transcripts (Fig. 6C) and the apparent accumulation of *GA2ox* at anthesis (Fig. 6E), which may reflect an increase in the respective activities. These results are schematically summarized in Fig. 8, obtained by the integration of RNA-seq and qRT–PCR data. The second consists of the rapid loss of calyptras after bloom, and partially of stamens (Fig. 6A), in which transcripts supporting the synthesis of GA are highly abundant (*VvGA20ox1*, *VvGA20ox 2*, *VvGA3ox2*, and *VvGA3ox3*, Fig. 4A, B). Even if the levels of bioactive GAs decrease in inflorescence after anthesis, it is proposed that their synthesis carries on in the carpel upon fruit-set, as suggested by the unchanging level of the *VvGA20ox3* transcript (abundant in the carpel). This scenario is similar to the one observed in other species: GA accumulation in fertilized ovaries coincides with *GA20ox* expression in tomato (Olimpieri *et al.*, 2007; Serrani *et al.*, 2007), and with transient up-regulation of GA20ox and GA3ox in *Arabidopsis* (Dorcey *et al.*, 2009).

In grapevine, the accumulation of GA_1_ only at anthesis may be explained by a stronger GA13ox function at this time, and/or by the involvement of *VvGA20ox1*, *VvGA20ox3*, and *VvGA3ox2*, whose gene products showed a partial activity in the early 13-hydroxylation pathway *in vitro*, or by genes missed by the present analysis.

The only up-regulated biosynthetic gene during fruit-set is the low abundant *VvGA3ox1*, whose expression in the inflorescence is mainly confined to the rachis (Figs 4B, 6D), a tissue discarded in the analysis in Fig. 6. This suggests a restricted expression to a limited number of cells in the inflorescence.

Stamens are a known site of active GA biosynthesis in several species (Itoh *et al.*, 1999, 2001; Hu *et al.*, 2008) where they play a role in stamen elongation and pollen maturation (Cheng *et al.*, 2004; Rieu *et al.*, 2008), and are also transported to tissues in their proximity. In *Arabidopsis*, GA biosynthesis is sustained in stamens (in both filaments and anthers) and pollen by the presence of high levels of different *GA20ox* and *GA3ox* transcripts (Hu *et al.*, 2008; Plackett *et al.*, 2012). A similar scenario is found in grapevine stamens where *VvGA20ox2*, *VvGA3ox2*, and *VvGA3ox3* are abundant (Figs 4A, B, 8).

In contrast to other species, however, where active GAs are suggested to accumulate in petals due to transport from stamens (Weiss and Halevy, 1989; Hu *et al.*, 2008), active GAs may accumulate in the grapevine calyptra by active synthesis, as suggested by the elevated expression of a number of GA metabolism genes in this organ (Fig. 4). The mechanisms that regulate calyptra detachment in grapevine are unknown, but may involve GAs, perhaps through regulation of cell expansion.

### GAs control development of different grapevine organs

Berry enlargement occurs mainly prior to véraison and is determined by rapid cell division followed by a phase of cell expansion, and a role for GA may be expected in both phases. Indeed, previous reports on seeded and seedless cultivars showed that active GAs peak at anthesis, decrease after anthesis, peak again ~10–20 d after anthesis (Pérez *et al.*, 2000), and then decrease during berry development. In agreement with this previous work, the present data show that transcripts of enzymes involved in biosynthesis are abundant in flowers but low in berries at the very early stages of development.

Developing seeds are a known source of GAs in many plant species (Singh *et al.*, 2002), whereas seeds approaching maturation are rich in GA-deactivating activities probably to avoid premature germination (Thomas, *et al.*, 1999; Hedden and Thomas, 2012). In *Arabidopsis*, GAs are synthesized in seeds by *AtGA20ox* and *AtGA3ox2*–*AtGA3ox4*. *GA3ox* genes are expressed in developing seeds in different locations in the embryo, and GA synthesis in seeds partially contributes to the growth of the silique. In developing seeds, GA biosynthesis occurs upon fertilization. In seeded cultivars, grape seeds develop from 10 d to 20 d after anthesis at the pea-size stage (Coombe, 1960). Although it remains unclear which GAs accumulate in grape seeds due to a lack of reliable measurement and since no abundant *GA20ox* and *GA3ox* transcripts have been detected, some information can be inferred by comparing the present RNA-seq data with the qRT–PCR data of Fig. 4. A clue to the presence of GA in seeds is given by the elevated abundance of the *VvGA2ox4* transcript both in seeds and in pea-size berries, a tissue retaining the forming seed (Fig. 4C). Probably, the accumulation of GA deactivation mechanisms in the seeds selected for this study (from berries at the green-hard stage) is an indication of approaching maturity. Biosynthesis of GAs in seeds may involve *VvGA20ox2* which is expressed to a relevant level in pea-size berries (Fig. 4A).

Tendrils share the same meristematic origin as inflorescences in grapevine (Srinivasan and Mullins, 1979, 1981), and the decision regarding whether that meristem will become a tendril or an inflorescence depends on the GA response (Boss and Thomas, 2000, 2002). Metabolism of GAs in tendril may be fully understood by further characterization of *VvGA3ox1* and *VvGA2ox6* which are specifically expressed in this organ (Fig. 4B, D).

Similarly, a further study of GA metabolism in grapevine roots should consider the expression of *VvGA20ox3*, *VvGA3ox2*, and *VvGA2ox5* (Fig. 4A–C).

## Supplementary data

Supplementary data are available at *JXB* online.

Figure S1. Alignment of GA oxidases of grapevine and *A. thaliana*.

Figure S2. Gene structure of grapevine GA oxidase genes.

Figure S3. Phylogenetic analyses of GA oxidases.

Figure S4. *Arabidopsis* plants overexpressing grapevine *GA2ox* genes.

Table S1. Grapevine 2-ODDs analysed in this work.

Table S2. List of primers used in this work, their nucleotide sequences, and the experiment in which they were used.

Text S1. Parameters for GA identification in mass spectrometry.

Supplementary Data

## Abbreviations:

BS: bootstrap support
FA: formic acid
GA: gibberellin
GA2ox: gibberellin 2-oxidase
GA3ox: gibberellin 3-oxidase
GA20ox: gibberellin 20-oxidase
GST: glutathione *S*-transferase
MS/MS: tandem mass spectrometry
NRQ: normalized relative quantity
2-ODD: 2-oxoglutarate dependent dioxygenase
PP: posterior probability
qRT–PCR: quantitative reverse transcription–polymerase chain reaction
RNA-seq: whole transcriptome shotgun sequencing
RPKM: reads per kilobase per million mapped reads
SD: standard deviation
UPLC: ultra performance liquid chromatography.
