# Supplementary material for: Gibberellin metabolism in Vitis vinifera L. during bloom and fruit-set: functional characterization and evolution of grapevine gibberellin oxidases
Source: J Exp Bot. 2013 Sep 4;64(14):4403–19. doi: 10.1093/jxb/ert251 (PMC3808322; doi:10.1093/jxb/ert251)
Supplement: Supplementary Data [file supp_64_14_4403__index.html]

Gibberellin metabolism in Vitis vinifera L. during bloom and fruit-set: functional characterization and evolution of grapevine gibberellin oxidases — Supplementary Data 

# Gibberellin metabolism in *Vitis vinifera* L. during bloom and fruit-set: functional characterization and evolution of grapevine gibberellin oxidases

## Supplementary Data

Data files

**Files in this Data Supplement:**

- Supplementary Data - Supplementary Data
