# Supplementary material for: Gibberellin metabolism in Vitis vinifera L. during bloom and fruit-set: functional characterization and evolution of grapevine gibberellin oxidases
Source: J Exp Bot. 2013 Sep 4;64(14):4403–19. doi: 10.1093/jxb/ert251 (PMC3808322; doi:10.1093/jxb/ert251)
Supplement: Supplementary Data [file supp_ert251_Supplementary_data4.pdf]

Gibberellin metabolism in *Vitis vinifera* L. during bloom and fruit-set: functional characterization and evolution of grapevine gibberellin oxidases

RUNNING TITLE: Gibberellin metabolism in grapevine during fruit-set.

AUTHORS:
Giacomelli Lisa, Rota-Stabelli Omar, Masuero Domenico, Acheampong Atiako Kwame, Moretto Marco, Caputi Lorenzo, Vrhovsek Urska, and Moser Claudio

SUPPLEMENTARY DATA

FIGURE S1: Alignment of GA oxidases of grapevine and *A. thaliana*.
MUSCLE alignment of GA20ox (**A**), GA3ox (**B**), and GA2ox (**C**) proteins of grapevine and *A. thaliana*. Conserved residues are highlighted in color. Motifs common to 2-ODD proteins: HDH iron binding domain (yellow), RxS, and NyYPxCxxP residues involved in the binding of 2-oxoglutarate (red), (Xu, *et al*., 1995; Kang et al., 2002). Motifs specific for each functional class: a leucine residue essential for function in OsGA20ox2 (Spielmeyer, 2002) is present in all thre GA oxidases groups (cyan); LPWKET (green) suggested to be involved in the binding of the GA substrate in GA20ox (Xu, *et al*., 1995); the HYRAD MNTLD AFTNW and QpxHI PMQFI WPDEE K sequences typical of GA20ox proteins (Han and Zhu, 2011); FFCLP LHQKQ KAQRK PGEHC GYGSA FIGRF FSKLM WKETF S typical of GA20ox and absent in GA3ox proteins (Han and Zhu, 2011); SYRWG xPSAT SxxQx SxSEA FHixx xxxS and DVKxT GxKVG LxRFL I typical of C_{20}-GA2ox proteins: present in AtGA2ox7 and 8 (Shomburg, *et al*., 2003)

FIGURE S2: Gene structure of grapevine GA oxidase genes.
Exons are represented by filled rectangles and introns by lines. Scale is indicated in kilobase pairs. The genes whose structures were experimentally determined are indicated in bold. The gene structures of *VvGA20ox4*, *VvGA20ox5*, *VvGA20ox6* and *VvGA2ox8* are based on predictions.

FIGURE S3: Phylogenetic analyses of GA oxidases.
A) Maximum Likelihood tree using a PRANK alignment and the LG+G model.
Supports at nodes are BS supports from the analyses of 100 pseudo-replicates (under maximum likelihood) and Bayesian PP using PhyloBayes and the LG+G model. When not shown the support at nodes is equal to 100. The tree supports a sister relationship between GA3ox and C_{19}-GA2ox

(Group A). Most GA oxidases of *V. vinifera* (indicated in red) cluster in four well distinct subgroups.

B) Phylogenetic analysis using a MUSCLE alignment, LG+L maximum likelihood tree with Bayesian posterior probabilities at key nodes.

Grapevine proteins are indicated in red. Accession numbers: PttGA3ox1 (AAR12160); CmGA23ox (AAB64347); CmGA3ox (CAB92914); Ps2ox2 (AF100954); Ps2ox1 (AF100955); Ntc12 (AB016083.1); LeGA20ox1 (AF049898); LeGA20ox2 (AF049899); LeGA20ox3 (AF049900); SoGA2ox1 (AF506281); SoGA2ox2 (AF506282); Le3OH1 (AB010991); Le3OH2 (AB010992); Ls3h1 (AB012205); Ls3h2 (AB012206); SoGA3ox1 (AF506280); StGA3ox1 (ACN89835); StGA3ox2 (ACN89834); PsGA3ox (AAC96015); NTY (AB032198); HaGA20ox1 (CAQ43616); HaGA20ox2 (CAQ43617); ZmGA20ox5 (ACN25832); ZmGA20ox1 (NM_001112453); ZmGA20ox2 (NM_001143386); ZmGA20ox4 (NM_001156071); Ls20ox1 (AB012203); Ls20ox2 (AB012204); *G. max* and *O. sativa* sequences are indicated with names reported in (Han and Zhu, 2011). OsGA20ox6 and OsGA20ox7 do not cluster with known GA20ox proteins, thus their function should be tested.

FIGURE S4: *Arabidopsis* plants overexpressing grapevine *GA2ox* genes.

Six weeks old plants from three independent overexpressing lines (T3) per genotype are shown together with wild type Col-0 (top left). From the left: 35S::VvGA2ox7 lines X2.1 (top), A1.10.1 (middle) and Y1.1 (bottom); second column: 35S::VvGA2ox5 lines F1.1 (top), E9.1 (middle) and A1.6.1 (bottom); third column: 35S::VvGA2ox2 lines 3.1.4 (top), D1.1 (middle), and E3.4 (bottom); last column on the right: 35S::VvGA2ox3 lines A3.2 (top), D7.1 (middle), and B8.3 (bottom).

TABLE S1: Grapevine 2-ODDs analyzed in this work.

2-ODD protein names given in this work (column 1) are followed by their closest V1 (http://genomes.cribi.unipd.it/grape/) and V0 (www.genoscope.cns.fr/spip/Vitis-vinifera-e.html) gene prediction identifiers of the 12x *V. vinifera* genome, and by the translated coding sequence when experimentally determined.

TABLE S2: List of primers used in this work, their nucleotide sequences and the experiment in which they were used.

TEXT S1: Parameters for GA identification in Mass Spectrometry

Supplementary Figure S1: Alignment of grapevine and *A. thaliana* GA oxidases.

A GA20ox

```
                    5         15         25         35         45         55
AtGA20ox1  -----MAVSF VTTSPEEEDK PKLGLGNIQT PLIFNPSMLN LQAN------ IPNQFIWPDD
AtGA20ox2  ---MAILCTT TSPAEKEHEP KQDLEKDQTS PLIFNPSLLN LQSQ------ IPNQFIWPDE
AtGA20ox3  ---------M ATECIATVPQ IFSENKTKED SSIFDAKLLN QHSH-----H IPQQFVWPDH
AtGA20ox4  ---------- MECIIKLPQR FNKNKSKKNP LRIFDSTVLN HQPD-----H IPQEFVWPDH
AtGA20ox5  ------MCIY ASRQTVCPYL TPFKVKRPKS REMNSSDVNF SLLQ--SQPN VPAEFFWPEK
VvGA20ox1  MAMECCTSTM LMPPPRPPLK PLDEANTQHQ SLVFDASVLK YQST------ IPSQFIWPDE
VvGA20ox2  -------MDS SASTILMPPP LELKDERKKG SVVFDSSKMQ KQEK------ LPTEFIWPDA
VvGA20ox3  -----MSIVC VEGNPPSMFN PPTDDHKNQE PLVFDASVLR HQSN------ IPKQFIWPDA
VvGA20ox4  ---------- ------MASR TSTVVMAPPS EEKHDVGFLV FDPKKMAKQE LPKQFIWPNE
VvGA20ox5  ---------- ---------- -MALEIEEHP VESLSSGPKL CVKN-----F VWSEHEWPLI
VvGA20ox6  --------MD LGASTLLCPP LELTDKKEHG VSPSDSSFFK KQPN------ IPMEFIWAKG
```

typical of GA20ox

```
                   65         75         85         95        105        115
AtGA20ox1  EKPSINVLEL DVPLIDL-QN LLSDPSSTLD A-SRLISEAC KKHGFFLVVN HGISEELISD
AtGA20ox2  EKPSIDIPEL NVPFIDL--- --SSQDSTLE A-PRVIAEAC TKHGFFLVVN HGVSESLIAD
AtGA20ox3  EKPSTDVQPL QVPLIDLAGF LSGDSCLASE A-TRLVSKAA TKHGFFLITN HGVDESLLSR
AtGA20ox4  EKPSKNVPIL QVPVIDLAGF LSNDPLLVSE A-ERLVSEAA KKHGFFLVTN HGVDERLLST
AtGA20ox5  DV-APSEGDL DLPIIDLSGF LNGNEAETQL A-AKAVKKAC MAHGTFLVVN HGFKSGLAEK
VvGA20ox1  EKPCAKPPEL LVPPIDLGGF LSADPLAISN A-ARLVNEAC RKHGFFLVVN HGVDAQLVTE
VvGA20ox2  DL-VRAQQEL NEPLIDLDGF FKGDEAATAH A-AELIRMAC LNHGFFQVTN HGVDLDLIRA
VvGA20ox3  EKPGDKATEL SVPLIDLGGF LSGDPAAAME A-TRLVREAC QKHGFFLVVN HGVDDKLIYK
VvGA20ox4  DL-VEAKEEL NEPLVDMEGF LKGDEVATAH A-AELVRQAC VTHGFFQVIN HGVNPDILRA
VvGA20ox5  NH-DDFADGD DIPTISLEGN LSGKPCQDYD KVCQVMVTAC EKWGFFKLVD HGVAIETVEN
VvGA20ox6  EV-GHAHEEL REPVVDLEGF FSGDEVATQH A-AMLVRSAC LNHGFFQVIN HRVDPHLITV
```

```
                  125        135        145        155        165        175
AtGA20ox1  AHEYTSRFFD MPLSEKQRVL RKSGESVGYA SSFTGRFSTK LPWKETLSFR FCDDMS----
AtGA20ox2  AHRLMESFFD MPLAGKQKAQ RKPGESCGYA SSFTGRFSTK LPWKETLSFQ FSNDNS----
AtGA20ox3  AYLHMDSFFK APACEKQKAQ RKWGESSGYA SSFVGRFSSK LPWKETLSFK FSPEEK----
AtGA20ox4  AHKLMDTFFK SPNYEKLKAQ RKVGETTGYA SSFVGRFKEN LPWKETLSFS FSPTEKSENY
AtGA20ox5  ALEISSLFFG LSKDEKLRAY RIPGNISGYT AGHSQRFSSN LPWNETLTLA FKKGPP----
VvGA20ox1  AHKNMDFFFN MSLSEKQRAQ RKVGDHCGYA SSFTGRFSTK LPWKETLSFR YCDDDQ----
VvGA20ox2  AQEDMGAFFK LPLSRKLSVK KKPGELSGYS GAHADRYTSK LPWKETLSFV YCYDSG----
VvGA20ox3  AHQYMDSFFG LPLAKKQRAQ RKLGEHCGYA SSFIGRFSSK LPWKETLSFS YSAEKK----
VvGA20ox4  TEVGLDPYFN LPMSRKLSMH KKVDELCGYC GAHSDRFAAK LTWKEMLTFE YHFKGD----
VvGA20ox5  VKVQLNELFD LPMDQKLKGA RSTSLPLGYC ASNPE-YGQN LPWAEILQLL QSPQQV----
VvGA20ox6  AHDHMEAFFK LPWSKKLRAQ RKPGSLWGYS GAHADRFSLK LPWKETLSFG FHENGS----
```

typical of GA20ox and absent in GA3ox

```
                    185        195        205        215        225        235
AtGA20ox1    -RSKSVQDYF CDALGHGFQP FGKVYQEYCE AMSSLSLKIM ELLGLSLGV- KRDYFREFFE
AtGA20ox2    -GSRTVQDYF SDTLGQEFEQ FGKVYQDYCE AMSSLSLKIM ELLGLSLGV- NRDYFRGFFE
AtGA20ox3    IHSQTVKDFV SKKMGDGYED FGKVYQEYAE AMNTLSLKIM ELLGMSLGV- ERRYFKEFFE
AtGA20ox4    S--QTVKNYI SKTMGDGYKD FGSVYQEYAE TMSNLSLKIM ELLGMSLGI- KREHFREFFE
AtGA20ox5    ---HVVEDFL TSRLGNHRQE IGQVFQEFCD AMNGLVMDLM ELLGISMGLK DRTYYRRFFE
VvGA20ox1    -SSRIIEKYF SNVMGEDFKQ FGRVYQQYCE AMSRLSLGIM ELLGMSLGV- GREYFREFFE
VvGA20ox2    -SKPMVADYF KTALGEDFEQ IGWIYQKYCD ALKELSLGIM QLLAISLDV- DSSYYRKLFE
VvGA20ox3    -SSNAVQEYF LNKMGEDFSE FGQVYQDYCE AMSTLSLVIM ELLGMSLGI- GGAHFREFFE
VvGA20ox4    -TDSEVADYF KNVVGEDFEE -TWVFQRFCE AMKDLSLKIF KLLAISLNLE DTSYCEKFFE
VvGA20ox5    ------VAFA RKVFGDQHQP FSNAMVKYMQ ALDKLGMKIF EMLAHGMGL- PDDFFTKNFE
VvGA20ox6    --ESVVEDFF KSTLGEEFEQ TGLVYQKYCQ AMKDLSLVLM ELLAISLGV- DRLHYRKFFE

                    245        255        265        275        285        295
AtGA20ox1    END-SIMRLN YYPPCIKPDL TLGTGPHCDP TSLTILHQDH VNGLQVF-VE NQWRSIRPNP
AtGA20ox2    END-SIMRLN HYPPCQTPDL TLGTGPHCDP SSLTILHQDH VNGLQVF-VD NQWQSIRPNP
AtGA20ox3    DSD-SIFRLN YYPQCKQPEL ALGTGPHCDP TSLTILHQDQ VGGLQVF-VD NKWQSIPPNP
AtGA20ox4    DNE-SIFRLN YYPKCKQPDL VLGTGPHCDP TSLTILQQDQ VSGLQVF-VD NQWQSIPPIP
AtGA20ox5    DGS-GIFRCN YYPPCKQPEK ALGVGPHNDP TAITVLLQDD VVGLEVF-AA GSWQTVRPRP
VvGA20ox1    GND-SIMRLN YYPPCQKPNL TLGTGPHCDP TSLTILHQDQ VSGLQVF-VD EKWHSISPNS
VvGA20ox2    DGY-SIMRCN SYPPCKEAGL VMGTGPHCDP VALTILHQDQ VKGLEVF-VD NKWQSVKPRP
VvGA20ox3    END-SIMRLN YYPPCLKPDL TLGTGPHCDP TSLTILHQDQ VGGLQVF-VD DKWWSISPNF
VvGA20ox4    DGF-AVLRCN YYPVCPESGL TFGTGPHCDP TSLTILHQDQ VGGLEVF-SN EKWYYVKPQP
VvGA20ox5    EKEATMIRVN RYPPCPLPEK CLGVGSHSDP HTLTILLQDD VGGLQVLKSD NQWIGIRPVP
VvGA20ox6    DGS-SIMRCN YYPPCQEPGL AFGTGPHCDP TSLTILHQDQ VGGLEVY-VN NKWRTIRPRC

                    305        315        325        335        345        355
AtGA20ox1    KAFVVNIGDT FMALSNDRYK SCLHRAVVNS ESERKSLAFF LCPKKDRVVT PPRELL-DS-
AtGA20ox2    KAFVVNIGDT FMALSNGIFK SCLHRAVVNR ESARKSMAFF LCPKKDKVVK PPSDIL-EK-
AtGA20ox3    HAFVVNIGDT FMALTNGRYK SCLHRAVVNS ERERKTFAFF LCPKGEKVVK PPEELV-NG-
AtGA20ox4    QALVVNIGDT LMALTNGIYK SCLHRAVVNG ETTRKTLAFF LCPKVDKVVK PPSELE----
AtGA20ox5    GALVVNVGDT FMALSNGNYR SCYHRAVVNK EKVRRSLVFF SCPREDKIIV PPPELVEGE-
VvGA20ox1    EAFVVNIGDT FMALSNGIYK SCLHRAVVNS QTPRKSLAFF LCPKMEKVVS PPNGLV-DA-
VvGA20ox2    GALVVNIGDT FMALSNGKYK SCIHRAVVNM DKERRSLTFF MSPKDDKVVS PPQELI-VR-
VvGA20ox3    DAFVVNIGDT FMALSNGRYK SCLHRAVVNS QTPRKSLAFF LCPEKDKVVR PPTELV-DT-
VvGA20ox4    GALVINIGDT LMALTNGIYK SCLHRAAAKN DRDRISFAYF ISPRQDKLVS PIKDLI-AR-
VvGA20ox5    NSFVINIGDT LEAWTNGRLR SVVHRAVVNK EKHRLSVAYF LSPATSAIID CPPQLI-ESS
VvGA20ox6    DALVINLGDT FKALSNGRYK SCLHRAVVNR YKERRSLVFF VCPRENKVVT PPQDLV-CR-

                    365        375        385        395        405
AtGA20ox1    ----ITSRRY PDFTWSMFLE FTQKHYR-AD MNTLQAFSDW LTKPI-----
AtGA20ox2    ----MKTRKY PDFTWSMFLE FTQKHYR-AD VNTLDSFSNW VITNNNPI--
AtGA20ox3    --VKSGERKY PDFTWSMFLE FTQKHYR-AD MNTLDEFSIW LKNRRSF---
AtGA20ox4    ----G-ERAY PDFTWSMFLE FTMKHYR-AD MNTLEEFTNW LKNKGSF---
AtGA20ox5    ----EASRKY PDFTWAQLQK FTQSGYR-VD NTTLHNFSSW LVSNSDKKST
VvGA20ox1    ----NNPRIY PDFTWSSLLE FTQKHYR-AD TKTLHVFSNW LQQKNN----
VvGA20ox2    ----EGPRKY PDFKWSELLE FTQKHYR-PN NDTLQSFVEW RLSSQTK---
VvGA20ox3    ----NSPRIY PDFTWSNLLE FTQKHYR-AD MKTLEVFSSW LQQKTAEAV-
VvGA20ox4    ----EGSKKY PDFKWEQLLD FTQVHDR-TD DTTIKRFVEF LESSETK---
VvGA20ox5    TN----LRKY VSFTWGEFRK ELLTQKRVVG KTALNRYLIS P---------
VvGA20ox6    ----EGTRKY PDFKWSDLLE FTQKYYR-AD DATLQNFTKW LLSSNPPNHH
                                 __________________
                                  typical of GA20ox
```

B **GA3ox**

```
                  5         15         25         35         45         55
AtGA3ox1    MPAMLTDVFR GHPIHLPHSH IPDFTSLREL PDSYKWTPKD D---LLFSAA PSPPATGENI
AtGA3ox2    MSSTLSDVFR SHPIHIPLSN PPDFKS---L PDSYTWTPKD D-------LL FSASASDETL
AtGA3ox3    -MSSVTQLFK NNPVNRDRII PLDFTNTKTL PDSHVWSKPE P--------- ---ETTSGPI
AtGA3ox4    ---------- -MPSLAEEIC IGNLGSLQTL PESFTWKLTA ADSLLRPSSA VSFDAVEESI
VvGA3ox1    -MPSRISDAF KAHPLHLNHR HLDLNSVQEL PDLYAWAGVD E-------NP SGDSLITESV
VvGA3ox2    MASTLSQVFR DNPLPLNHII PLDFTSVHSL PESHVWPAFD G-----FPFG TTYPGEKFSI
VvGA3ox3    -MPSELSDAF KSMPANLYKK QLDLNSIQEL PDSHAWASLG E-------HP CVDSLIAESV

                 65         75         85         95        105        115
AtGA3ox1    PLIDLDHPD- ATNQIGHACR TWGAFQISNH GVPLGLLQDI EFLTGSLFGL PVQRKLKSAR
AtGA3ox2    PLIDLSDIH- VATLVGHACT TWGAFQITNH GVPSRLLDDI EFLTGSLFRL PVQRKLKAAR
AtGA3ox3    PVISLSNPE- EHGLLRQACE EWGVFHITDH GVSHSLLHNV DCQMKRLFSL PMHRKILAVR
AtGA3ox4    PVIDLSNPD- VTTLIGDASK TWGAFQIANH GISQKLLDDI ESLSKTLFDM PSERKLEAAS
VvGA3ox1    PVIDLTDPN- ASELVGHACK SWGVFQVTNH GIPGSLLDDI ESAGRSLFSL PAQQKLKAAR
VvGA3ox2    PIIDLMDPN- AAQLVGHACE KWGAFQLTSH GLPSILTDDV ESQTRRLFAL PAHEKMKALR
VvGA3ox3    PVIDLSDPN- ALTLVGDACK SWGVFQVINH GIPISLLEAI EDASRNLFAL PAEQKLKATR

                125        135        145        155        165        175
AtGA3ox1    SETGVSGYGV ARIASFFNKQ MWSEGFTITG SPLND-FRKL WPQHH-LNYC DIVEEYEEHM
AtGA3ox2    SENGVSGYGV ARIASFFNKK MWSEGFTVIG SPLHD-FRKL WPSHH-LKYC EIIEEYEEHM
AtGA3ox3    SPDESTGYGV VRISMFYDKL MWSEGFSVMG SSLRRHATLL WPDDH-AEFC NVMEEYQKAM
AtGA3ox4    SDKGVSGYGE PRISPFFEKK MWSEGFTIAD DSYRNHFNTL WPHDH-TKYC GIIQEYVDEM
VvGA3ox1    SPDGVAGYGL ARISSFFNKL MWYEGFTIFG SPLEH-ARQL WPQDY-TKFC DVTEEFEKEM
VvGA3ox2    LPSGGTGYGQ ARISPFYPKF MWHEGFTIMG SAVDH-ARKL WPDDY-KGFC DVMEDYQKKM
VvGA3ox3    PPDGFSGFGQ PRIAPFFAKQ MWYEGFTVLG SPLEL-VSKL WPEEYCTKFC EVTEEYDKQM

                185        195        205        215        225        235
AtGA3ox1    KKLASKLMWL ALNSLGVSEE DIEWASLSSD LN-----WAQ AALQLNHYPV CPEPDRAMGL
AtGA3ox2    QKLAAKLMWF ALGSLGVEEK DIQWAGP-NS DFQG---TQ- AVIQLNHYPK CPEPDRAMGL
AtGA3ox3    DDLSHRLISM LMGSLGLTHE DLGWLVPDKT GSGT--DSIQ SFLQLNSYPV CPDPHLAMGL
AtGA3ox4    EKLASRLLYC ILGSLGVTVE DIEWAHKLEK SGSK---VGR GAIRLNHYPV CPEPERAMGL
VvGA3ox1    NQLAERLMWL MLGSLGITKE DLNWAGSKGD FK-------- AALQLNSYPA CPEPDRAMGL
VvGA3ox2    KELAESLLHI FLESLDI-SK E----EYRST TIQRGREACN TALQLNSYPP CPDPNRAMGL
VvGA3ox3    KQLANKLLWL LLGSLGINKE DVEWAGP-EG QLEG---AH- AALQLNSYPA CPQPDKAMGL

                245        255        265        275        285        295
AtGA3ox1    AAHTDSTLLT ILYQNNTAGL QV---FRDDL GWVTVPPFPG SLVVNVGDLF HILSNGLFKS
AtGA3ox2    AAHTDSTLMT ILYQNNTAGL QV---FRDDV GWVTAPPVPG SLVVNVGDLL HILTNGIFPS
AtGA3ox3    APHTDSSLLT ILYQGNIPGL EIESPQEEGS RWIGVEPIEG SLVVIMGDLS HIISNGQFRS
AtGA3ox4    AAHTDSTILT ILHQSNTGGL QV---FREES GWVTVEPAPG VLVVNIGDLF HILSNGKIPS
VvGA3ox1    AAHTDSSLFT ILYQNTVSGL QV---QREGA GWITVPPLPG ALVINVGDLL HILSNGVFPS
VvGA3ox2    APHTDSLLFT IVHQSHTSGL QI---LRDGV GWITVFPLEG ALVVNVGDLL HILSNGRYPS
VvGA3ox3    AEHTDSSLLT ILYQGSTSGL QV--VL-EGS GWITVPPLPG ALVVNIGDLL HILSNAAFPS

                305        315        325        335        345        355
AtGA3ox1    VLHRARVNQT RARLSVAFLW -GPQSDIKIS PVPKLVSP-- -VESPLYQSV TWKEYLRTKA
AtGA3ox2    VLHRARVNHV RSRFSMAYLW -GPPSDIMIS PLPKLVDP-- -LQSPLYPSL TWKQYLATKA
AtGA3ox3    TMHRAVVNKT HHRVSAAYFA -GPPKNLQIG PLTSDK---- -NHPPIYRRL IWEEYLAAKA
AtGA3ox4    VVHRAKVNHT RSRISIAYLW GGPAGDVQIA PISKLTGP-- -AEPSLYRSI TWKEYLQIKY
VvGA3ox1    VVHRALVNRT KHRLSVAYLY -GPPAGVPIS PVPKLVDS-- -THPPLYRPV TWSEYLCTKA
VvGA3ox2    VLHRAVVNQA EHRISLAYFY -GPPADSLIS PLCNLVSSGQ QVVAPRYRSV SVKEYVDLKE
VvGA3ox3    VLHRAMVNNS KQRISVAYFY -GPPATIPVA PIPKLVDS-- -SHPPVYRSV TWSEFLATKA
```

```
                    365       375       385
AtGA3ox1     THFNKALSMI RNHREE---- ---------- --
AtGA3ox2     THFNQSLSII RN-------- ---------- --
AtGA3ox3     THFNKALTLF RC-------- ---------- --
AtGA3ox4     EVFDKAMDAI RV-VNPTN-- ---------- --
VvGA3ox1     KHFDKALSLV RL-CMPRNGF IDVNDHNGVK VG
VvGA3ox2     KHKEKALSLL RL-------- ---------- --
VvGA3ox3     KHFNKALSLV RM-PVPETDS SE-------- --
```

C GA2ox

```
                      5         15         25         35         45         55
AtGA2ox1     ---------- ----MAVLSK PVA------I PKS------- ---------- GFSLIPVIDM
VvGA2ox1     -MVVLSKPTI ADFPPIINCK STT------- ---------- ---------- LFPVIPTVDL
VvGA2ox2     MVVLSQNGRQ QLPLIKACKP NYG------- ---------- ---------- -FTGIPVIDM
VvGA2ox3     MGVFSKPA-- --IEQLPLIR NCM------- ---------- ---------- PFSGIPLIDL
VvGA2ox4     ---------- MVVASPNPIG SEK------- ---------- ---------L IAVEVPIIDL
VvGA2ox5     ---------- MVVPSPSPIR SKK------- ---------- ---------T KAVGIPVIDL
AtGA2ox2     MVVLPQPV-- TLDNHISLIP TYK-----PV PVL------- ---------- TSHSIPVVNL
AtGA2ox3     MVIVLQPASF DSNLYVNPKC KPR------- ---------- ---------- -PVLIPVIDL
AtGA2ox4     ---------- -------MVK GSQ------- ---------- ------KIVA VDQDIPIIDM
AtGA2ox6     --------MV LPSSTPLQTT GKK------- ---------- ------TISS PEYNFPVIDF
AtGA2ox7     --MASQPPFK TNFCSIFGSS FPN-----ST SESNTNTSTI Q--------T SGIKLPVIDL
AtGA2ox8     ---MDPPFNE IYNNLLYNQI TKKDNDVSEI PFSFSVTAVV E--------- -EVELPVIDV
VvGA2ox6     --MESEPPFG EFVNSVFGNI VEQ------- ---------- -EETEAKFDV DECELPLIDL
VvGA2ox7     --MDSDPPFE ETYKTLFENS IEE------- --SKINRANQ I-----LITC EECELPLIDI
VvGA2ox8     --MTNSNPPL LQHYGVLCQH GET------- PAVQQRDCGN N-----GAAM EECQLPLIDL
```

```
                     65         75         85         95        105        115
AtGA2ox1     -----SDPE- ---SKHALVK ACEDFGFFKV INHGVSAELV SVLEHETVDF FSLPKSEKTQ
VvGA2ox1     -----SEPDS ----KHLVVK SCEEFGFFKV INHGIPLELI SRLETEVIEF FSLSLSEKQK
VvGA2ox2     -----TSPH- ---AKAHMVE ACEEFGFFKL VNHGVPMEFM SRLEDEGIKF FSLPQSEKDL
VvGA2ox3     -----SQPD- ---SKALLIE ACQEFGFFKV INHGVPMDLI SKLEAEAINF FSLPLSEKEK
VvGA2ox4     -------AAR GPKVAELIVK ASQEYGFFKV INHGVPEDVI RKMEEESFNF FGKPDSEKKK
VvGA2ox5     -------SLN RSAIAELIVN ACEDYGFFKV VNHGVPKEII GRLEEEGLSF FAKPSSEKQK
AtGA2ox2     -----ADPE- ---AKTRIVK ACEEFGFFKV VNHGVRPELM TRLEQEAIGF FGLPQSLKNR
AtGA2ox3     -----TDSD- ---AKTQIVK ACEEFGFFKV INHGVRPDLL TQLEQEAINF FALHHSLKDK
AtGA2ox4     -------SQE RSQVSMQIVK ACESLGFFKV INHGVDQTTI SRMEQESINF FAKPAHEKKS
AtGA2ox6     ------SLND RSKLSEKIVK ACEVNGFFKV INHGVKPEII KRFEHEGEEF FNKPESDKLR
AtGA2ox7     SHLTSGEEVK RKRCVKQMVA AAKEWGFFQI VNHGIPKDVF EMMLLEEKKL FDQPFSVKVR
AtGA2ox8     SRLIDGAEEE REKCKEAIAR ASREWGFFQV INHGISMDVL EKMRQEQIRV FREPFDKKSK
VvGA2ox6     -GHLNLGNLE HEECKRKICE ASTEWGFFQI VNHGVSKEIL SRIHREQVEL FRQPFQIKTN
VvGA2ox7     -GRLSMGELE REECKKEIAR ASQEWGFFQV INHGVSSEIL EDMRSKQMQV FKQPFRLKTN
VvGA2ox8     EGLWSESEEE RLACASAIGR ASSKWGFFQV VNHGIRPELL SEMRREQVKL FETPFERKAA
```

```
                125        135        145        155        165        175
AtGA2ox1   VAG-----YP FG---YGNSK IGRNGDVGWV EYLLMNANHD SG---SGPLF PSLLKSPGTF
VvGA2ox1   AGPP----DP FG---YGNRS IGPNGDVGWV EYLLLTMNQE RNSQKLATIF G---KYPEKL
VvGA2ox2   AGPP----QP FG---YGNRR IGPNGDVGWI EYLLLSTNPD SISQICSSIS P---ENPEIF
VvGA2ox3   AGPP----NP SG---YGNKR IGSSGDIGRV EYLLLNPQSF PS------VF G---QNPDMF
VvGA2ox4   AGPA----QP FG---YGCKN IGFNGDMGEV EYLLFNTNPH CI-SQRSETI S---NDPTKF
VvGA2ox5   AGPA----SP FG---YGCKN IGFNGDRGEL EYLLLHTNPV SI-SERSKAI S---NDPTEF
AtGA2ox2   AGPP----EP YG---YGNKR IGPNGDVGWI EYLLLNANPQ LSSPKTSAVF R---QTPQIF
AtGA2ox3   AGPP----DP FG---YGTKR IGPNGDLGWL EYILLNANLC LESHKTTAIF R---HTPAIF
AtGA2ox4   VRPVN---QP FR---YGFRD IGLNGDSGEV EYLLFHTNDP A----FRSQL S--------F
AtGA2ox6   AGPA----SP FG---YGCKN IGFNGDLGEL EYLLLHANPT AVADKSETIS H---DDPFKF
AtGA2ox7   ERFSDLSKNS ---YRWGNPS ATSPAQYSVS EAFHIILSEV SR-------I S---DDRNNL
AtGA2ox8   SEKFS-A-GS ---YRWGTPS ATSIRQLSWS EAFHVPMTDI SD-------- N---KDFTTL
VvGA2ox6   EKLLN---LS SGCYRWGTQT AITQKQFAWS EAFHIPLSTI FQ-------- ---LSEFEGL
VvGA2ox7   HQYLN---LS AGCYRWGTPT ATCLSQLSWS EAFHIPLMDI SS----SGGL P------TTL
VvGA2ox8   CQLLD---NS ---YRWGTPT ATCPKELSWS EAFHIPLTKV SE----EACY G----EFCSL
```

typical of C_{20}-GA2ox

```
                185        195        205        215        225        235
AtGA2ox1   RNALEEYTTS VRKMTFDVLE KITDGL-GIK PRN----TLS KLVSDQ-NTD SILRLNHY-P
VvGA2ox1   CSALNDYVLA VKKMACELLE LMADGL-RIK PRN----VFS KLLMDE-QSD SVFRLNHY-P
VvGA2ox2   GSAVVDYVSS VKNMTYEVLE MIAEGL-NIE PRN----VLS RLLKDE-ESD SCFRVNHY-P
VvGA2ox3   RSAVSDYLSA VRKMACEILE LLADGL-MIQ PRN----VFS KLLMDE-QSD SVFRLNHY-P
VvGA2ox4   SSAVSGYIQA VTDLACEILD LMAEGL-WV- QNT----SVF SSLITHLDSD SVFRLNHYPP
VvGA2ox5   SCAVTDYIQG VRELCCEILD LIGEGL-WL- QDKMVFSRMI RDVHSD---- SVIRVNHY-P
AtGA2ox2   RESVEEYMKE IKEVSYKVLE MVAEEL-GIE PRD----TLS KMLRDE-KSD SCLRLNHY-P
AtGA2ox3   REAVEEYIKE MKRMSSKFLE MVEEEL-KIE PKE----KLS RLVKVK-ESD SCLRMNHY-P
AtGA2ox4   SSAVNCYIEA VKQLAREILD LTAEGL-HV- PPH----SFS RLISSV-DSD SVLRVNHY-P
AtGA2ox6   SSATNDYIRT VRDLACEIID LTIENLWGQ- -KSSEVSELI RDVRSD---- SILRLNHY-P
AtGA2ox7   RTIVETYVQE IARVAQMICE ILGKQV-NV- -SS----EYF ENIFEL-EN- SFLRLNKYHP
AtGA2ox8   SSTMEKFASE SEALAYMLAE VLAEKS-GQ- -NS----SFF KENCVR-NT- CYLRMNRY-P
VvGA2ox6   RSSLQEFAIK ASDLAQQIAK ILAENL-GC- -KS----TFF FKNCLP-SS- CYIRMNRY-P
VvGA2ox7   SSTMGQFAAT VSDLAQRLVE ILAEEM-GH- -KS----TFF KEKCLP-ST- CYIRMNRY-P
VvGA2ox8   REVMQEFAKA MSNLARLLAG VLAESL-GH- -QK----GVF DEICDE-NT- CFLRLNRY-P
```

```
                245        255        265        275        285        295
AtGA2ox1   PCPLS----- -NKKTNGGKN VI-----GFG EHTDPQIISV LRSNNTSGLQ INLNDGS--W
VvGA2ox1   PYSEL----- ---QASNGKN MI-----GFG EHTDPQIISV LRSNNTSGLQ ISLGNGS--W
VvGA2ox2   PCPEL----- ---EALRGRV LV-----GFG EHTDPQIISV LRSNNTNGLE ICLKDGT--W
VvGA2ox3   PYPER----- ---QALSGKC MI-----GFG EHTDPQIISV LRSNNTSGLQ ISLRNGN--W
VvGA2ox4   PLKDRDTSSS SSFQIHHQGN NI-----GFG EHSDPQILTI LRSNDVGGLQ ICLGDGV--W
VvGA2ox5   AVKDV----- ---KEWDPCD PI-----GFG EHSDPQILTI LRSNDVPGLQ IRLRDGL--W
AtGA2ox2   AAEEE----- ---------AE KM--VKVGFG EHTDPQIISV LRSNNTAGLQ ICVKDGS--W
AtGA2ox3   EKEET----- ------PVKE EI-----GFG EHTDPQLISL LRSNDTEGLQ ICVKDGT--W
AtGA2ox4   PSDQF----- -FGEANLSDQ SVSLTRVGFG EHTDPQILTV LRSNGVGGLQ VSNSDGM--W
AtGA2ox6   PAPYA----- -----LSGVG QI-----GFG EHSDPQILTV LRSNDVDGLE ICSRDGL--W
AtGA2ox7   SVFGS----- ---------E VF-----GLV PHTDTSFLTI LSQDQIGGLE L---ENNGQW
AtGA2ox8   PCPKP----- ---------SE VY-----GLM PHTDSDFLTI LYQDQVGGLQ L-IKDNR--W
VvGA2ox6   ACPVS----- ---------SK VF-----GLI PHTDSDFLTV LHQDQVGGLQ L-LKDGK--W
VvGA2ox7   PCPTS----- ---------Q IF-----GLM PHTDSDFLTI LHQDQVGGLQ L-VKDGR--W
VvGA2ox8   PCPVS----- ---------PE VF-----GLV PHTDSDFLTI LYQDEVGGLQ L-MKDSK--W
```

```
                 305        315        325        335        345        355
AtGA2ox1    ISVPPDHTSF FFNVGDSLQV MTNGRFKSVR HRVLANCKK- SRVSMIYFAG PSLTQRIAPL
VvGA2ox1    ISVPPDANSF FINVGDSLQV MTNGRFKSVR HRVLANSIK- SRISMIYFGG PPLNEKIAPL
VvGA2ox2    VSVPPDQDSF FINVGDSLQV MTNGKFKSVK HRVVTESRK- ARVSMIYFGG PPLTEIIAPL
VvGA2ox3    ISVPPDENSF FINVGDSLQV MTNGRFQSVK HRVLTNSCK- SRVSMIYFGG PPLSEKIAPL
VvGA2ox4    VPVPPDPTSF CVNVGDLLQA MTNGRFVSVR HRALTNSDK- PRMSMAFFGA PPLHALITSP
VvGA2ox5    VPVPPDPTEF CVFVGDALEA MTNGRLMSVR HRALTSSVK- ARLSMMYFGA PPLNAWISPL
AtGA2ox2    VAVPPDHSSF FINVGDALQV MTNGRFKSVK HRVLADTRR- SRISMIYFGG PPLSQKIAPL
AtGA2ox3    VDVTPDHSSF FVLVGDTLQV MTNGRFKSVK HRVVTNTKR- SRISMIYFAG PPLSEKIAPL
AtGA2ox4    VSVSPDPSAF CVNVGDLLQV MTNGRFISVR HRALTYGEE- SRLSTAYFAG PPLQAKIGPL
AtGA2ox6    IPIPSDPTCF FVLVGDCLQA LTNGRFTSVR HRVLANTAKK PRMSAMYFAA PPLEAKISPL
AtGA2ox7    ISVKPCLEAL TVNIGDMFQA LSNGVYQSVR HRVIS-PANI ERMSIAFFVC PYLETEIDCF
AtGA2ox8    IAVKPNPKAL IINIGDLFQA WSNGMYKSVE HRVMTNPKV- ERFSTAYFMC PSYDAVIECS
VvGA2ox6    IRVKPNPDAL VINIGDLFQA WSNGVYKSLE HRVVANHEI- ERFSFAYFLC PSHDTVIQSC
VvGA2ox7    IAVKPNPEAL IINIGDLFQA WSNGVYKSVQ HRVVTNQKV- ERFSTAYFLC PSYDAVIESC
VvGA2ox8    VAVKPNKDTL IVNIGDLFQA WSNNEYKSVE HQVMANAIK- ERYSIAYFLC PSYDAFIGSC
```

```
                 365        375        385        395        405
AtGA2ox1    TCLIDN-EDE RLYEEFTWSE Y-KNSTYNSR LSDNRLQQ-- FERKTIKNLL N---
VvGA2ox1    PSLV--EGKE SLYKEFTWFE Y-KRSAYKSR LGDNRLSQ-- FERTAAT--- ----
VvGA2ox2    PSLVKE-GEE TLYKEFTWAD Y-KKSVYKSR LADNRLGL-- FEKKEQP--- ----
VvGA2ox3    PSLM--EGEE SHYKEFTWFE Y-KRSAYNTR LADNRLVF-- FQKVAAT--- ----
VvGA2ox4    PEVVTP-ERP SLYRPFTWAE Y-KEITYSLR LGDSRLNL-- FKACPDKEAQ E---
VvGA2ox5    PDMVSP-QKP SLYRPFSWVE Y-KKAAYSLR LGDRRLDL-- FKINTSSTGD KVEL
AtGA2ox2    PCLVPE-QDD WLYKEFTWSQ Y-KSSAYKSK LGDYRLGL-- FEKQPLLNHK TLV-
AtGA2ox3    SCLVPK-QDD CLYNEFTWSQ Y-KLSAYKTK LGDYRLGL-- FEKRPPFSLS NV--
AtGA2ox4    SAMVMTMNQP RLYQTFTWGE YKKRAYSL-R LEDSRLDM-- FRTCKD---- ----
AtGA2ox6    PKMVSP-ENP RRYNSFTWGD Y-KKATYSLR LDVPRLEF-- FKTL------ ----
AtGA2ox7    -------GYP KKYRRFSFRE YKEQSEHDVK ETGDKVGL-- -SRFLI---- ----
AtGA2ox8    SD-------R PAYRNFSFRE FRQQVQEDVK KFGFKVGL-- -PRFLNHVY- ----
VvGA2ox6    CE-------P SIYRKFSFRE YRQQVEEDVK TTGDKVGL-- -SRFRR---- ----
VvGA2ox7    VEPL------ -LYRKFSFRE FRQQVQEDVQ KLGYKVGL-- -PRFLV---- ----
VvGA2ox8    SE-------P TIYRKFTFGE YRQQVQEDVK KTGHKVGL-- -PRFLLQTTQ ----
```

typical of C_{20}-GA2ox

Supplementary Figure S2

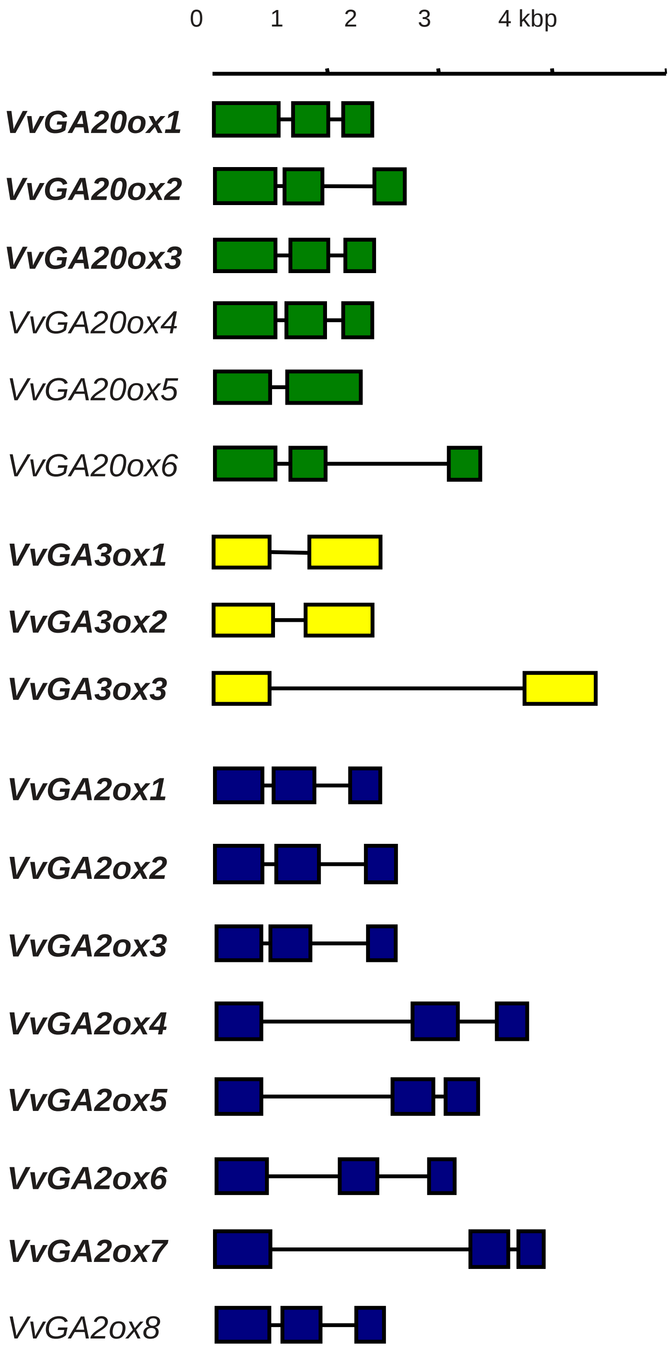

Supplementary Figure S3A

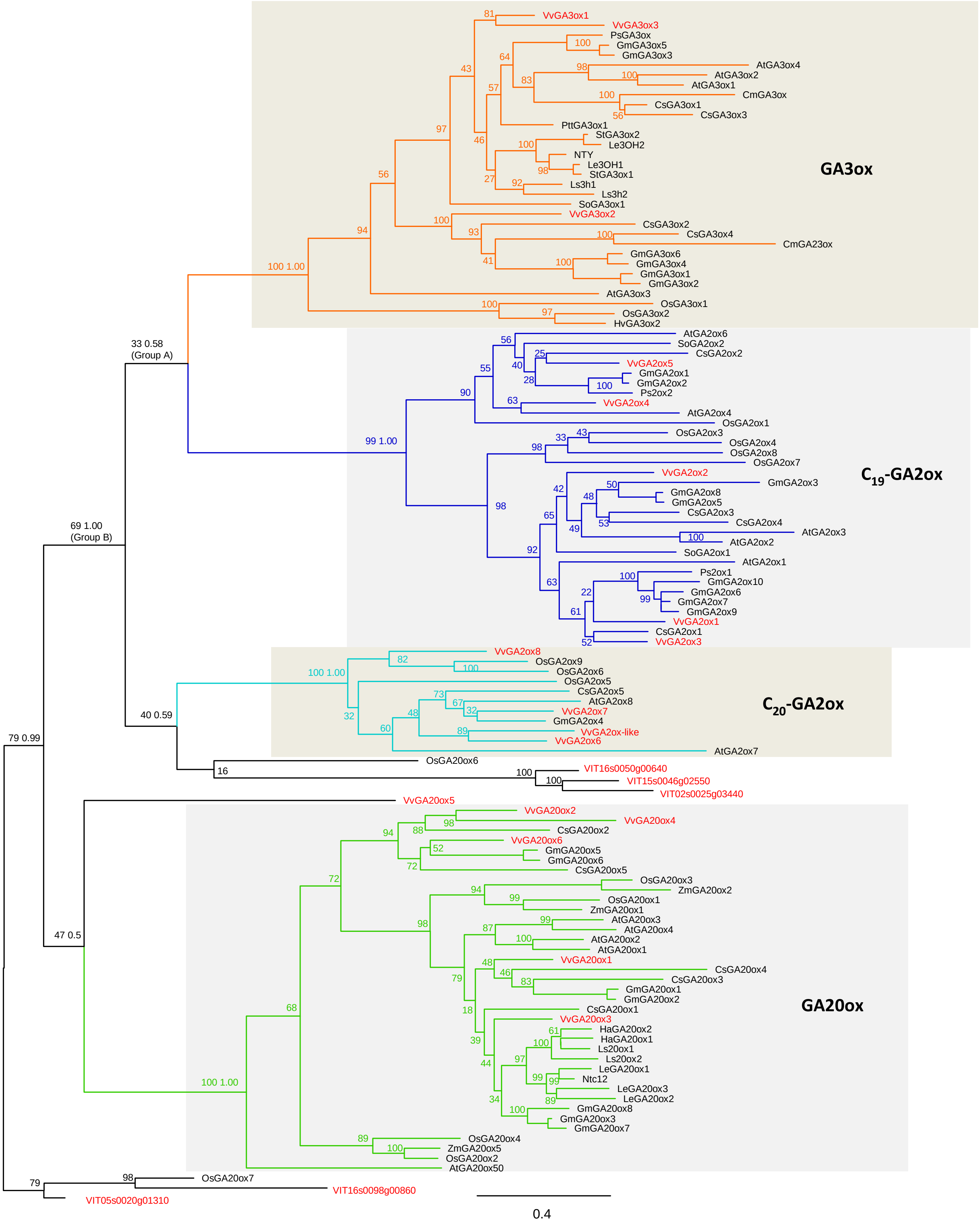

Supplementary Figure S3B

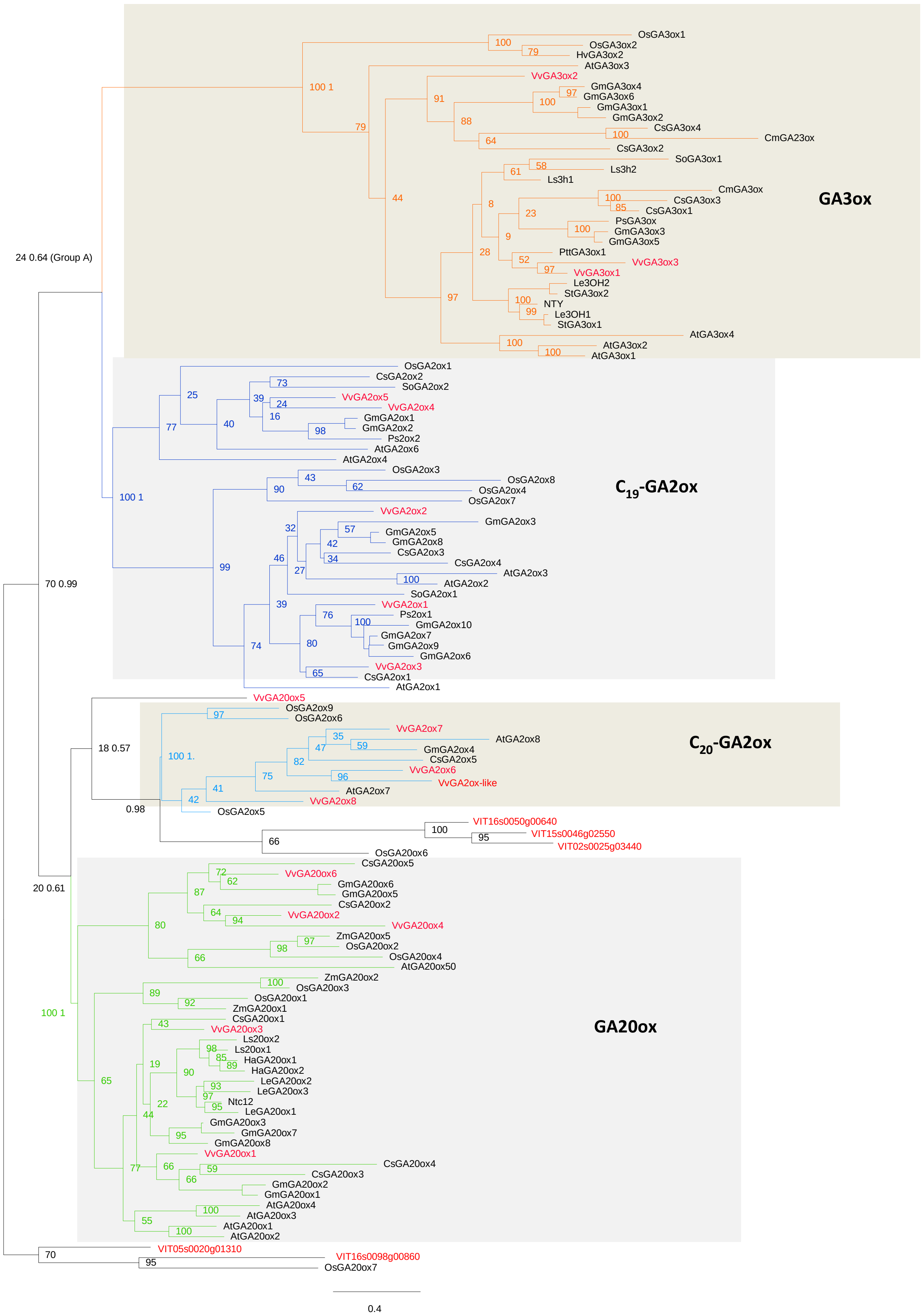

Supplementary Figure S4

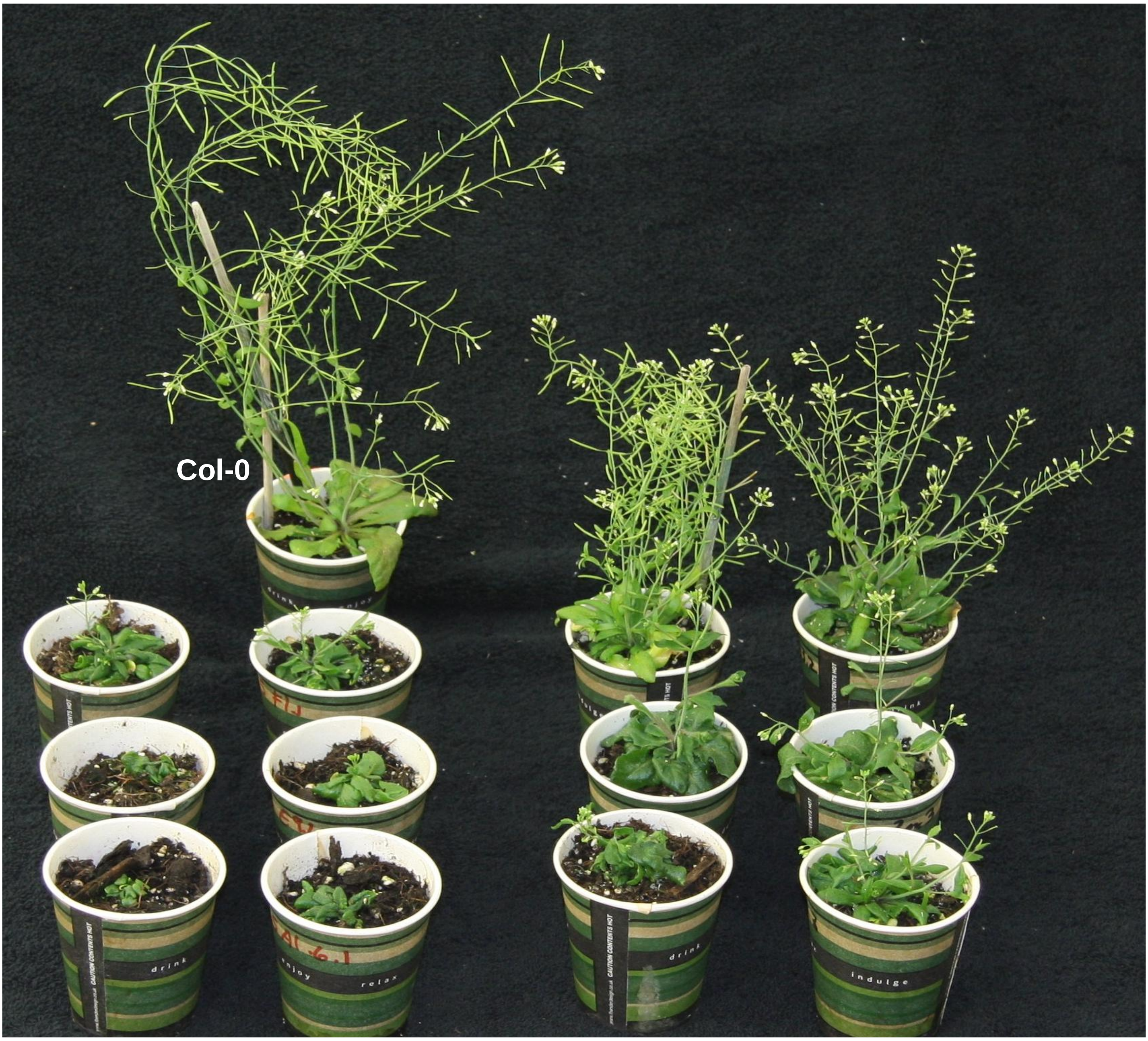

35S::VvGA2ox7 **35S::VvGA2ox5** **35S::VvGA2ox2** **35S::VvGA2ox3**

Supplementary Table S1

Grapevine 2-oxoglutarate dependent dioxygenases analyzed in this work: names given in this work (column 1) are followed by GenBank accession numbers, their closest gene prediction identifiers derived from the V1 (http://genomes.cribi.unipd.it/grape/) and V0 (http://www.genoscope.cns.fr/spip/Vitis-vinifera-e.html) predictions of the 12x genome of *V. vinifera*, and by the translated coding sequence when experimentally determined. Genes whose full-length sequences were cloned from cDNA and verified by sequencing are indicated in bold, and their GenBank accession number is indicated. *VvGAox-like* genes were not analyzed for they form a separate cluster in our phylogenetic analysis.

Name	GenBank Acc. No.	Prediction V1	Prediction V0	Comment	Amino acid sequence
**VvGA3ox1**	KC898176	VIT_09s0002g05270	GSVIVT01017173001		MPSRISDAFKAHPLHLNHRHLDLNSVQELPDLYAWAGVDENPSGDSLITESVPVIDLTDPNASELVGHACKSWGVFQVTNHG<br>IPGSLLDDIESAGRSLFSLPAQQKLKAARSPDGVAGYGLARISSFFNKLMWYEGFTIFGSPLEHARQLWPQDYTKFCDVTEEF<br>EKEMNQLAERLMWLMLGSLGITKEDLNWAGSKGDFKAALQLNSYPACPEPDRAMGLAAHTDSSLFTILYQNTVSGLQVQRE<br>GAGWITVPPLPGALVINVGDLLHILSNGVFPSVVHRALVNRTKHRLSVAYLYGPPAGVPISPVPKLVDSTHPPLYRPVTWSEYL<br>CTKAKHFDKALSLVRLCMPRNGFIDVNDHNGVKVG
**VvGA3ox2**	KC898175	VIT_04s0008g04940	GSVIVT01035796001		MASTLSQVFRDNPLPLNHIIPLDFTSVHSLPESHVWPAFDGFPFGTTYPGEKFSIPIIDLMDPNAAQLVGHACEKWGAFQLTSH<br>GLPSILTDDVESQTRRLFALPAHEKMKALRLPSGGTGYGQARISPFYPKFMWHEGFTIMGSAVDHARKLWPDDYKGFCDVM<br>EDYQKKMKELAESLLHIFLESLDISKEEYRSTTIQRGREACNTALQLNSYPPCPDPNRAMGLAPHTDSLLFTIVHQSHTSGLQIL<br>RDGVGWITVFPLEGALVVNVGDLLHILSNGRYPSVLHRAVVNQAEHRISLAYFYGPPADSLISPLCNLVSSGQQVVAPRYRSV<br>SVKEYVDLKEKHKEKALSLLRL
**VvGA3ox3**	KC898177	VIT_09s0002g05300	GSVIVT01017178001		MPSELSDAFKSMPANLYKKQLDLNSIQELPDSHAWASLGEHPCVDSLIAESVPVIDLSDPNALTLVGDACKSWGVFQVINHGI<br>PISLLEAIEDASRNLFALPAEQKLKATRPPDGFSGFGQPRIAPFFAKQMWYEGFTVLGSPLELVSKLWPEEYCTKFCEVTEEY<br>DKQMKQLANKLLWLLLGSLGINKEDVEWAGPEGQLEGAHAALQLNSYPACPQPDKAMGLAEHTDSSLLTILYQGSTSGLQV<br>VLEGSGWITVPPLPGALVVNIGDLLHILSNAAFPSVLHRAMVNNSKQRISVAYFYGPPATIPVAPIPKLVDSSHPPVYRSVTWS<br>EFLATKAKHFNKALSLVRMPVPETDSSE
**VvGA2ox1**	KC898179	VIT_19s0140g00140	GSVIVT01000689001	Activity not tested	MVVLSKPTIADFPPIINCKSTTLFPVIPTVDLSEPDSKHLVVKSCEEFGFFKVINHGIPLELISRLETEVIEFFSLSLSEKQKAGPPD<br>PFGYGNRSIGPNGDVGWVEYLLLTMNQERNSQKLATIFGKYPEKLCSALNDYVLAVKKMACELLELMADGLRIKPRNVFSKLL<br>MDEQSDSVFRLNHYPPYSELQASNGKNMIGFGEHTDPQIISVLRSNNTSGLQISLGNGSWISVPPDANSFFINVGDSLQVMTN<br>GRFKSVRHRVLANSIKSRISMIYFGGPPLNEKIAPLPSLVEGKESLYKEFTWFEYKRSAYKSRLGDNRLSQFERTAAT
**VvGA2ox2**	KC898180	VIT_10s0003g03490	GSVIVT01021468001		MVVLSQNGRQQLPLIKACKPNYGFTGIPVIDMTSPHAKAHMVEACEEFGFFKLVNHGVPMEFMSRLEDEGIKFFSLPQSEKD<br>LAGPPQPFGYGNRRIGPNGDVGWIEYLLLSTNPDSISQICSSISPENPEIFGSAVVDYVSSVKNMTYEVLEMIAEGLNIEPRNVL<br>SRLLKDEESDSCFRVNHYPPCPELEALRGRVLVGFGEHTDPQIISVLRSNNTNGLEICLKDGTWVSVPPDQDSFFINVGDSLQ<br>VMTNGKFKSVKHRVVTESRKARVSMIYFGGPPLTEIIAPLPSLVKEGEETLYKEFTWADYKKSVYKSRLADNRLGLFEKKEQP
**VvGA2ox3**	KC898181	VIT_19s0140g00120	GSVIVT01000687001		MGVFSKPAIEQLPLIRNCMPFSGIPLIDLSQPDSKALLIEACQEFGFFKVINHGVPMDLISKLEAEAINFFSLPLSEKEKAGPPNP<br>SGYGNKRIGSSGDIGRVEYLLLNPQSFPSVFGQNPDMFRSAVSDYLSAVRKMACEILELLADGLMIQPRNVFSKLLMDEQSD<br>SVFRLNHYPPYPERQALSGKCMIGFGEHTDPQIISVLRSNNTSGLQISLRNGNWISVPPDENSFFINVGDSLQVMTNGRFQSV<br>KHRVLTNSCKSRVSMIYFGGPPLSEKIAPLPSLMEGEESHYKEFTWFEYKRSAYNTRLADNRLVFFQKVAAT
**VvGA2ox4**	KC898182	VIT_05s0077g00520	GSVIVT01034945001		MVVASPNPIGSEKLIAVEVPIIDLAARGPKVAELIVKASQEYGFFKVINHGVPEDVIRKMEEESFNFFGKPDSEKKKAGPAQPFG<br>YGCKNIGFNGDMGEVEYLLFNTNPHCISQRSETISNDPTKFSSAVSGYIQAVTDLACEILDLMAEGLWVQNTSVFSSLITHLDS<br>DSVFRLNHYPPPLKDRDTSSSSSFQIHHQGNNIGFGEHSDPQILTILRSNDVGGLQICLGDGVWVPVPPDPTSFCVNVGDLLQ<br>AMTNGRFVSVRHRALTNSDKPRMSMAFFGAPPLHALITSPPEVVTPERPSLYRPFTWAEYKEITYSLRLGDSRLNLFKACPDK<br>EAQE
**VvGA2ox5**	KC898183	VIT_07s0005g01920	GIDVvT00037860001		MVVPSPSPIRSKKTKAVGIPVIDLSLNRSAIAELIVNACEDYGFFKVVNHGVPKEIIGRLEEEGLSFFAKPSSEKQKAGPASPFG<br>YGCKNIGFNGDRGELEYLLLHTNPVSISERSKAISNDPTEFSCAVTDYIQGVRELCCEILDLIGEGLWLQDKMVFSRMIRDVHS<br>DSVIRVNHYPAVKDVKEWDPCDPIGFGEHSDPQILTILRSNDVPGLQIRLRDGLWVPVPPDPTEFCVFVGDALEAMTNGRLM<br>SVRHRALTSSVKARLSMMYFGAPPLNAWISPLPDMVSPQKPSLYRPFSWVEYKKAAYSLRLGDRRLDLFKINTSSTGDKVEL
**VvGA2ox6**	KC898185	VIT_19s0177g00030	GSVIVT01001966001	Activity not tested	MTAALLGLLLLNVLWCAHGRYYVYDQIMTDQPLRKPVRMESEPPFGEFVNSVFGNIVEQEETEAKFDVDECELPLIDLGHLNL<br>GNLEHEECKRKICEASTEWGFFQIVNHGVSKEILSRIHREQVELFRQPFQIKTNEKLLNLSSGCYRWGTQTAITQKQFAWSEA<br>FHIPLSTIFQLSEFEGLRSSLQEFAIKASDLAQQIAKILAENLGCKSTFFFKNCLPSSCYIRMNRYPACPVSSKVFGLIPHTDSDF<br>LTVLHQDQVGGLQLLKDGKWIRVKPNPDALVINIGDLFQAWSNGVYKSLEHRVVANHEIERFSFAYFLCPSHDTVIQSCCEPSI<br>YRKFSFREYRQQVEEDVKTTGDKVGLSRFRR

**VvGA2ox7**	KC898184	VIT_10s0116g00410	GSVIVT01012628001		MVESDTKLPFSMDSDPPFEETYKTLFENSIEESKINRANQILITCEECELPLIDIGRLSMGELEREECKKEIARASQEWGFFQVINHGVSSEILEDMRSKQMQVFKQPFRLKTNHQYLNLSAGCYRWGTPTATCLSQLSWSEAFHIPLMDISSSGGLPTTLSSTMGQFAATVSDLAQRLVEILAEEMGHKSTFFKEKCLPSTCYIRMNRYPPCPTSQIFGLMPHTDSDFLTILHQDQVGGLQLVKDGRWIAVKPNPEALIINIGDLFQAWSNGVYKSVQHRVVTNQKVERFSTAYFLCPSYDAVIESCVEPLLYRKFSFREFRQQVQEDVQKLGYKVGLPRFLV
VvGA2ox8		VIT_01s0010g01650	GSVIVT01010228001	Full length cDNA not detected	
**VvGA20ox1**	KC898188	VIT_15s0048g01320	GSVIVT01027572001		MAMECCTSTMLMPPPRPPLKPLDEANTQHQSLVFDASVLKYQSTIPSQFIWPDEEKPCAKPPELLVPPIDLGGFLSADPLAISNAARLVNEACRKHGFFLVVNHGVDAQLVTEAHKNMDFFFNMSLSEKQRAQRKVGDHCGYASSFTGRFSTKLPWKETLSFRYCDDDQSSRIIEKYFSNVMGEDFKQFGRVYQQYCEAMSRLSLGIMELLGMSLGVGREYFREFFEGNDSIMRLNYYPPCQKPNLTLGTGPHCDPTSLTILHQDQVSGLQVFVDEKWHSISPNSEAFVVNIGDTFMALSNGIYKSCLHRAVVNSQTPRKSLAFFLCPKMEKVVSPPNGLVDANNPRIYPDFTWSSLLEFTQKHYRADTKTLHVFSNWLQQKNN
**VvGA20ox2**	KC898186	VIT_04s0044g01650	GSVIVT01026453001		MDSSASTILMPPPLELKDERKKGSVVFDSSKMQKQEKLPTEFIWPDADLVRAQQELNEPLIDLDGFFKGDEAATAHAAELIRMACLNHGFFQVTNHGVDLDLIRAAQEDMGAFFKLPLSRKLSVKKKPGELSGYSGAHADRYTSKLPWKETLSFVYCYDSGSKPMVADYFKTALGEDFEQIGWIYQKYCDALKELSLGIMQLLAISLDVDSSYYRKLFEDGYSIMRCNSYPPCKEAGLVMGTGPHCDPVALTILHQDQVKGLEVFVDNKWQSVKPRPGALVVNIGDTFMALSNGKYKSCIHRAVVNMDKERRSLTFFMSPKDDKVVSPPQELIVREGPRKYPDFKWSELLEFTQKHYRPNNDTLQSFVEWRLSSQTK
**VvGA20ox3**	KC898189, DQ508817 (Dauelsberg *et al*., 2011)	VIT_16s0022g02310	GIDVvT00040228001		MSIVCVEGNPPSMFNPPTDDHKNQEPLVFDASVLRHQSNIPKQFIWPDAEKPGDKATELSVPLIDLGGFLSGDPAAAMEATRLVREACQKHGFFLVVNHGVDDKLIYKAHQYMDSFFGLPLAKKQRAQRKLGEHCGYASSFIGRFSSKLPWKETLSFSYSAEKKSSNAVQEYFLNKMGEDFSEFGQVYQDYCEAMSTLSLVIMELLGMSLGIGGAHFREFFEENDSIMRLNYYPPCLKPDLTLGTGPHCDPTSLTILHQDQVGGLQVFVDDKWWSISPNFDAFVVNIGDTFMALSNGRYKSCLHRAVVNSQTPRKSLAFFLCPEKDKVVRPPTELVDTNSPRIYPDFTWSNLLEFTQKHYRADMKTLEVFSSWLQQKTAEAV
VvGA20ox4		VIT_04s0044g01520	GSVIVT01026466001	Full length cDNA not detected	
VvGA20ox5		VIT_02s0234g00010	GSVIVT01004790001	Full length cDNA not detected	
VvGA20ox6		VIT_18s0001g01390	GSVIVT01008782001	Full length cDNA not detected	
**Additional not expressed or non functional genes**					
VvGA2ox-like		VIT_19s0177g00020	GSVIVT01001965001	Pseudogene or truncated	
**Vv2ODD-like1**	KC898178	VIT_05s0020g01310	GSVIVT01017737001	Not a GA oxidase	MAPVPISQIKVGHIDDVQELRKSIPAAIPERFIRDMAERPTLTMETLLSSTDIPVIDFSQLLKGKTDELQRELSKLAASCEEWGFFQVINHGIDLGLLESIEKAAMDFFMLPLEEKQKYAMAPGTIQGYGQAFVFSEDQKLDWCNMFALGLEPHFIRNPKLWPTKPAEFSDALEVYSREIRELCQNMLRYIAMSLGLNEDAFENMFGEAVQAVRMNYYPPCSRPDLVLGLSPHSDGSALTVLQQGRDGSVGLQILRHNTWVPVRPIPNAFVINIGDTIEVLTNGKYKSVEHRAVTHKEKDRLSIVTFYAPSYEIELGPMPEFLDENNPCKYRRYNHGEYSRHYVTSKLEGKKTLDFAKIHTNSSS
**Vv2ODD-like2**	KC898187	VIT_16s0098g00860	GSVIVT01038652001	Not a GA oxidase	MESKVLSTGIRYLTLPQSYIRPEPERPRLSQVSECKHVPIIDLGKDVNRAQLIQHIADACRLYGFFQVINHGVAAEMMEKMLEVADEFYRLPVEEKMKLYSDDPTKTMRLSTSFNVNKEKVHNWRDYLRLHCYPLDQYTPEWPSNPPSFKEIVSSYCKEVRELGFRLQEMISESLGLEKDHIKNVFGEQGQHMAVNYYPPCPQPELTYGLPGHTDPNALTILLQDLRVAGLQVLKDGTWLAIKPHPGAFVVNIGDQLQAVSNGKYKSVWHRAVVNAESERLSVASFLCPCNDAVIGPAKPLTEDGSAPIYKNFTYAEYYKKFWGRDLDQEHCLELFKN
VvGAox-like		VIT_15s0046g02550		Not analyzed	
VvGAox-like		VIT_16s0050g00640		Not analyzed	
VvGAox-like		VIT_02s0025g03440		Not analyzed	

Supplementary Table S2

List of primers used in this work, their nucleotide sequences and the experiment in which they were used.

Primer name	SEQUENCE FORWARD PRIMER	SEQUENCE REVERSE PRIMER	Experiment
VvGA2ox1	TGCCAACTCCTTCTTCATCA	CCAAAACCCTATGCCTCACA	real time qRT-PCR
VvGA2ox1	GCAAATCCACCACATTATTCC	CCGTCCTCTCAAACTGGCTA	study of gene structure
VvGA2ox1	CACCATGGTGGTCTTGTCGAAACCAAC	CTATGTGGCGGCCGTCCTCT	cloning in pDEST15
VvGA2ox2	AGAGCACACAGACCCACAGA	GAGACCCAAGTCCCATCCTT	real time qRT-PCR
VvGA2ox2	GAATGGTCGTCAGCAGTTG	TTTTCTCAAAGAGCCCAAGC	study of gene structure
VvGA2ox2	CACCATGGTTGTGTTATCGCAGAATGG	TCATGGCTGCTCTTTTTTCTCAAAG	cloning in pDEST15
VvGA2ox3	TGTGAAGCATAGGGTGTTGA	ATGAGTGAGGGCAATGGTG	real time qRT-PCR
VvGA2ox3	GGGTGTCTTCTCTAAACCAGCA	AATGGCTCTCCTCTCCTTCC	study of gene structure
VvGA2ox3	CACCATGGGTGTCTTCTCTAAACCAG	TCATGTGGCTGCCACTTTCTG	cloning in pDEST15
VvGA2ox4	GCTCTGCTGTGAGTGGTTACA	ATGTGTGATGAGGCTGCTGA	real time qRT-PCR
VvGA2ox4	CAGCTAGGGGACCGAAGGT	CACTCTTGAGCTTCCTTGTCC	study of gene structure
VvGA2ox4	CACCATGGTGGTGGCCTCTCCAAC	TCACTCTTGAGCTTCCTTGTCCG	cloning in pDEST15
VvGA2ox5	ATGGCTTTTTCAAGGTGGTT	CTTCTGCTTCTCCGAACTGG	real time qRT-PCR
VvGA2ox5	TGGTGGTTCCTTCTCCATCT	GCGGCGGTCTCCTAATCT	study of gene structure
VvGA2ox5	CACCATGGTGGTTCCTTCTCCATCTC	TCATAGTTCAACTTTGTCTCCAGTAC	cloning in pDEST15
VvGA2ox6	GGGGCTTCTTCCAGATTGTC	AACTCCACTTGCTCACGATG	real time qRT-PCR
VvGA2ox6	TGGAATCAGAGCCACCATTT	TTGAAAGCCCTACCTTGTCC	study of gene structure
VvGA2ox6	CACCATGGAATCAGAGCCACCATTTG	TCATCTGCGAAATCTTGAAAGC	cloning in pDEST15
VvGA2ox7	GAATGAACCGATACCCACCA	TCCACCTCCCATCTTTGACT	real time qRT-PCR
VvGA2ox7	ACTCGGACCCACCATTTGA	CATCCTCTTGAACCTGCTGTC	study of gene structure
VvGA2ox7	ATGGTGGAGAGTGATACTAAGC	TTATACAAGAAACCTTGGGAGA	study of gene structure
VvGA2ox7	CACCATGGACTCGGACCCACCAT	TTATACAAGAAACCTTGGGAGA	cloning in pDEST15
VvGA2ox8	TGAGTGAGATGAGGAGAGAGCA	TTGAGACGAGTAGCAGTTGGAG	real time qRT-PCR
VvGA2ox8	ATGACCAACTCAAACCCACCT	GCAACTCCCTATGAAAGCATC	study of gene structure
VvGA2ox8	CACCATGACCAACTCAAACCCACCT	CTATTGTGTGGTCTGAAGCAGAAA	study of gene structure
VvGA20ox1	AGAAGCCCTGTGCCAAGC	GACAAGACGAGCCGCATTT	real time qRT-PCR
VvGA20ox1	CCTCCCCTGAAGCCCCTA	TTGAAGCCAGTTTGAGAAGACA	study of gene structure
VvGA20ox1	CACCATGGCAATGGAGTGTTGCACTT	TCAGTTGTTTTTCTGTTGAAGCC	cloning in pDEST15
VvGA20ox2	AGGATGGCTTGCTTGAACC	CCAGGCTTCTTCTTGACACTG	real time qRT-PCR
VvGA20ox2	AAGGAAGTGTGGTTTTTGACTC	TTTGAGATGAGAGACGCCATT	study of gene structure
VvGA20ox2	CACCATGGACTCGAGTGCCTCAACTAT	TTATTTGGTTTGAGATGAGAGACGC	cloning in pDEST15
VvGA20ox3	GCCTAAAACCCGACCTCACT	GGACCACCATTTGTCATCTACA	real time qRT-PCR

VvGA20ox3	ATGTCTATAGTTTGTGTCGAAGG	TTACACCGCTTCAGCTGTTTTC	study of gene structure
VvGA20ox3	CACCATGTCTATAGTTTGTGTCGAAGG	TTACACCGCTTCAGCTGTTTTC	cloning in pDEST15
VvGA20ox4	ACCCACGGCTTCTTCCAA	CCAATCCAACCTCTGTAGCA	real time qRT-PCR
VvGA20ox4	CCATCGGAGGAGAAGCAT	TTGGGTGAAGTCTAAAAGTTGTTC	study of gene structure
VvGA20ox4	CACCATGGCATCAAGAACCTCAACTGTT	TTATTTCGTTTCCGATGACTCAAGG	cloning in pDEST15
VvGA20ox5	GGGGCTTCCTGATGACTTCT	TGTTAGGGTATGGGGGTCTG	real time qRT-PCR
VvGA20ox5	AGGCCCCAAGCTATGTGTTA	GAGGGCAGTTTTACCCACAA	study of gene structure
VvGA20ox6	GGTGTTCTTCGTGTGCCCTA	CTTCCTTGTCCCTTCTCTGC	real time qRT-PCR
VvGA20ox6	GCTCCTGTGCCCTCCATT	CGAAGCAATCAACACTCTCTT	study of gene structure
VvGA3ox1	CCTCAGAACTCGTGGGTCAT	TGGGCAGGTAGAGAGAAAAGG	real time qRT-PCR
VvGA3ox1	ACCCTCTCCACCTCAATCAC	GGGCATACACAGCCGAACTA	study of gene structure
VvGA3ox1	CACCATGCCTTCGAGAATCTCTGATG	CTAACCTACTTTCACCCCATTG	cloning in pDEST15
VvGA3ox2	CCCCACATACAGACTCATTGC	TCCACCCAACTCCATCTCTC	real time qRT-PCR
VvGA3ox2	CCTCCGTTCATTCTCTACCTG	TGCCTTCTCCTTGTGCTTTT	study of gene structure
VvGA3ox2	CACCATGGCTTCTACTCTTTCCCAAG	TCATAATCTTAACAAAGAGAGTGCC	cloning in pDEST15
VvGA3ox3	TCCTCTGGCTCCTGCTTG	CCCTTCTAACTGACCTTCTGG	real time qRT-PCR
VvGA3ox3	CACTGGGAATCAACAAAGAAGA	GGCATCCTAACTAATGAAAGAGC	study of gene structure
VvGA3ox3	CACCATGCCTTCAGAACTCTCTGAT	TTACTCCGATGAATCCGTTTC	cloning in pDEST15
2ODD-like1	CACCATGGCTCCAGTACCCATTTCT	TTAAGATGAGCTATTGGTATGAATC	cloning of VIT_05s0020g01310 in pDEST15
2ODD-like2	CACCATGGAATCAAAGGTGTTGTCCAC	CTAGTTCTTGAATAGTTCCAAGCAA	cloning of VIT_16s0098g00860 in pDEST15
ACTIN	TCCTTGCCTTGCGTCATCTAT	CACCAATCACTCTCCTGCTACAA	real time qRT-PCR
EF1-alpha	GAACTGGGTGCTTGATAGGC	AACCAAAATATCCGGAGTAAAAGA	real time qRT-PCR
GAPDH	TTCTCGTTGAGGGCTATTCCA	CCACAGACTTCATCGGTGACA	real time qRT-PCR
SAND	CAACATCCTTTACCCATTGACAGA	GCATTTGATCCACTTGCAGATAAG	real time qRT-PCR
TUBULIN	TGTTGGTGAAGGCATGGAGG	AGATGACACGCCTGCTGAACT	real time qRT-PCR
UBIQUITIN	CCCTGGCGGACTACAACATC	TCAAATGGCTGAGACCCACA	real time qRT-PCR

SUPPLEMENTARY TEXT S1: Parameters for GA identification in Mass Spectrometry

GA identification by Mass Spectrometry was performed in multiple reaction monitoring mode considering the following transitions: m/z 347.2 >273.2 for GA_1, 345.1 >239.1 for GA_3, 331.1 >57.1 for GA_4, 363.2 >275.2 for GA_8, 315.0 >271.0 for GA_9, 331.3 >269.3 for GA_{12}, 331.1 >287.0 for GA_{20}, 347.2 >259.2 for GA_{29}, 347.2 >259.2 for GA_{34}, 331.1 >243.2 for GA_{51}, 347.3 >189.2 for GA_{53} 349.0 >275.0, 349.0 >287.0 for $^2H_2$-GA_1, 347.0 >241.0 for $^2H_2$-GA_3, 333.0 >245.0, 333.0 >259.0 for $^2H_2$-GA_4, 365.0 >259.0, 365.0 >276.0 for $^2H_2$-GA_8, and 317.0 >273.0 for $^2H_2$-GA_9 respectively. Cone voltage and collision energy were 42 V and 22 V for GA_1, 34 V and 14 V for GA_3, 42 V and 24 V for GA_4, 38 V and 16 V for GA_8, 36 V and 20 V for GA_9, 48 V and 32 V for GA_{12}, 38 V and 18 V for GA_{20}, 36 V and 16 V for GA_{29}, 48 V and 16 V for GA_{34}, 36 V and 16 V for GA_{51}, and 50 V and 32 V for $GA_{53,}$ 42 V and 22 V for $^2H_2$-GA_1, 30 V and 16 V for $^2H_2$-GA_3, 40 V and 20 V for $^2H_2$-GA_4, 38 V and 18 V for $^2H_2$-GA_8, 36 V and 20 V for $^2H_2$-GA_9. Concentrations of endogenous GA_1, GA_3, GA_4, GA_8, and GA_9 were corrected for the recovery of their respective deuterated internal standards, whereas concentrations of GA_{20} and GA_{34} were corrected for the recovery of $^2H_2$-GA_4.
